# The Aβ(1–38) peptide is a negative regulator of the Aβ(1–42) peptide implicated in Alzheimer disease progression

**DOI:** 10.1038/s41598-020-80164-w

**Published:** 2021-01-11

**Authors:** Maa O. Quartey, Jennifer N. K. Nyarko, Jason M. Maley, Jocelyn R. Barnes, Maria A. C. Bolanos, Ryan M. Heistad, Kaeli J. Knudsen, Paul R. Pennington, Josef Buttigieg, Carlos E. De Carvalho, Scot C. Leary, Matthew P. Parsons, Darrell D. Mousseau

**Affiliations:** 1 Cell Signalling Laboratory, Department of Psychiatry, University of Saskatchewan, GB41 HSB, 107 Wiggins Rd., Saskatoon, SK S7N 5E5 Canada; 2 Saskatchewan Structural Sciences Centre, University of Saskatchewan, Saskatoon, SK Canada; 3 Division of BioMedical Sciences (Neurosciences), Memorial University of Newfoundland, St. John’s, NL Canada; 4 Department of Biology, University of Regina, Regina, SK Canada; 5 Department of Biology, University of Saskatchewan, Saskatoon, SK Canada; 6 Department of Biochemistry, Microbiology and Immunology, University of Saskatchewan, Saskatoon, SK Canada

**Keywords:** Neuroscience, Diseases of the nervous system, Alzheimer's disease, Alzheimer's disease

## Abstract

The pool of β-Amyloid (Aβ) length variants detected in preclinical and clinical Alzheimer disease (AD) samples suggests a diversity of roles for Aβ peptides. We examined how a naturally occurring variant, *e.g.* Aβ(1–38), interacts with the AD-related variant, Aβ(1–42), and the predominant physiological variant, Aβ(1–40). Atomic force microscopy, Thioflavin T fluorescence, circular dichroism, dynamic light scattering, and surface plasmon resonance reveal that Aβ(1–38) interacts differently with Aβ(1–40) and Aβ(1–42) and, in general, Aβ(1–38) interferes with the conversion of Aβ(1–42) to a β-sheet-rich aggregate. Functionally, Aβ(1–38) reverses the negative impact of Aβ(1–42) on long-term potentiation in acute hippocampal slices and on membrane conductance in primary neurons, and mitigates an Aβ(1–42) phenotype in *Caenorhabditis elegans*. Aβ(1–38) also reverses any loss of MTT conversion induced by Aβ(1–40) and Aβ(1–42) in HT-22 hippocampal neurons and *APOE* ε4-positive human fibroblasts, although the combination of Aβ(1–38) and Aβ(1–42) inhibits MTT conversion in *APOE* ε4-negative fibroblasts. A greater ratio of soluble Aβ(1–42)/Aβ(1–38) [and Aβ(1–42)/Aβ(1–40)] in autopsied brain extracts correlates with an earlier age-at-death in males (but not females) with a diagnosis of AD. These results suggest that Aβ(1–38) is capable of physically counteracting, potentially in a sex-dependent manner, the neuropathological effects of the AD-relevant Aβ(1–42).

## Introduction

There is significant heterogeneity in the composition of the β-amyloid (Aβ) peptide pool in preclinical (*e.g*., mouse) and clinical (*e.g.*, brain, CSF, blood) samples, and these N- and C-terminally truncated peptides have likely contributed to the contention regarding the exact role of Aβ peptides in the brain and in the periphery (discussed in^1^). The physiological Aβ(1–40) peptide accounts for ~ 90% of the pool, but the contribution of Aβ(1–42) increases significantly in the Alzheimer disease (AD) brain^2^ and likely reflects relative shifts in α-, β- and γ-secretase-mediated cleavage of the Amyloid Protein Precursor (APP)^3^. The additional two hydrophobic amino acids in Aβ(1–42) contribute to its toxicity and its conversion to a β-sheet-rich conformation that tends to aggregate as plaque^4^, and it is this retention of Aβ(1–42) as plaque in brain that underpins the biochemical rationale for using *decreases* in the CSF or plasma Aβ(1–42)/Aβ(1–40) ratio as a marker of imminent onset or progression of AD^5–7^. Biomarker studies have focused primarily on Aβ(1–42) and/or Aβ(1–40), although other Aβ length variants are attracting attention. There are reports that AD patients tend to generate longer forms such as Aβ(1–42) and Aβ(1–43), whereas cognitively intact individuals tend to generate Aβ(1–37), Aβ(1–38) and Aβ(1–40)^8–10^. It has also been suggested that longer variants tend to aggregate as plaques (*e.g.* AD-related pathology), while shorter variants might preferentially target the vasculature (*e.g.,* cerebral amyloid angiopathy)^11,12^. Brain levels of soluble Aβ(1–38) are increased in cases of early-onset/familial AD^12^ and in experimental AD-related amyloidosis^12–14^. While these soluble peptides might not be associated with a diagnosis of AD-dementia per se, they do appear to be associated with a steeper rate of late-life cognitive decline^15^. In keeping with this relationship with soluble peptides, it is suggested that lower plasma levels of both Aβ(1–38) and Aβ(1–42), if measured concurrently, could be better indicators of incident AD-dementia^16^.

Functionally, synthetic as well as brain-derived Aβ peptides can disrupt long-term potentiation (LTP)^17–20^, a mechanism for synaptic strengthening critical for learning and memory. Yet soluble extracts from AD brain do not consistently impair experimental LTP, which may reflect differences in the relative proportion of N- and C-terminally truncated peptides in the total pool. Indeed, shorter peptides, including Aβ(1–38) and Aβ(1–40), do not appear to exert any overt effect in this functional paradigm^21^ and even with evidence that shorter variants, such as Aβ(1–38), might alter the fibrillogenic behaviour of Aβ(1–42) and provide some neuroprotection against Aβ(1–42) in cell culture^22^, many Aβ length variants are still presumed to be neurotoxic or amyloidogenic.

Motivated by these observations, we decided to re-evaluate the ‘amyloidogenic’ properties of Aβ(1–38), Aβ(1–40), and Aβ(1–42). Rather than simply repeating selected physicochemical assays used elsewhere that invariably rely on ‘aged’ (*e.g.,* oligomeric and fibril-rich) Aβ preparations, we chose to focus on small, primarily mono-/dimeric species that would be the most prevalent and active forms of Aβ in the earlier stages of the neuroamyloidogenic process commonly associated with AD. We now confirm that, when studied in isolation, Aβ(1–38) exhibits a degree of aggregation potential and functional effect; however, when studied in a mixture, Aβ(1–38) exerts a negative regulatory role on the physicochemical behaviour of Aβ(1–42) and associated functional disruption. Furthermore, clinical relevance is suggested by the correlation between an earlier age-at-death and an increase in the *soluble* Aβ(1–42)/Aβ(1–38) ratio in cortical samples of males, but not females, with late-onset AD.

## Results

### Biophysical assays

We monitored secondary structures or aggregation potential of our Aβ peptide mixtures using established biophysical techniques, including AFM, ThT fluorescence, CD, DLS, and SPR.

**Figure 1:**
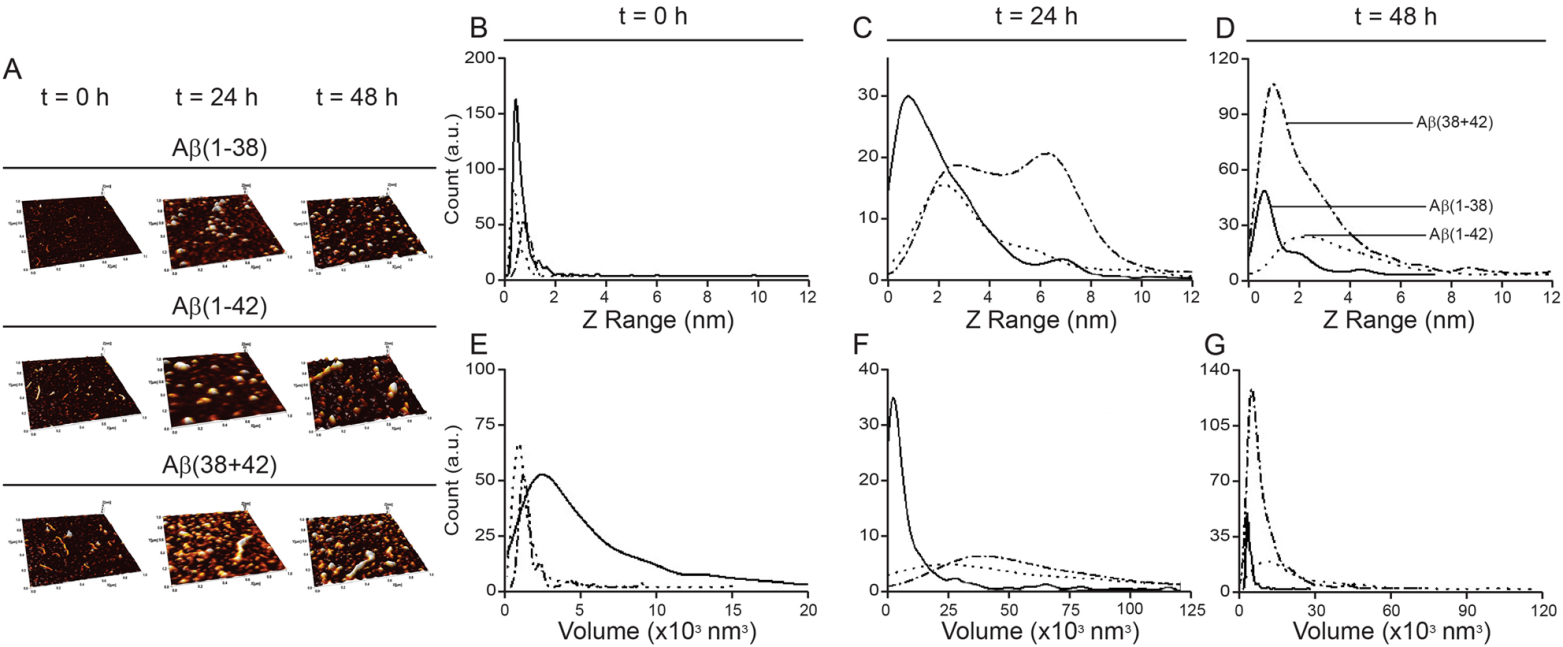
Aβ peptides incubate as aggregates with different properties in isolation or in a complex mixture. (**A**) AFM scans of surfaces used to measure the length (**B–D**) and volume (**E–G**) of aggregates derived from 20 µM solutions of the peptides alone, *e.g.* Aβ(1–38) (solid line) and Aβ(1–42) (dotted line), or a 20:1 mix of Aβ(1–42):Aβ(1–38) (dotdash line) at the zero ‘0′ time-point and at 24 and 48 h of incubation.

AFM measurements (Fig. 1A–G) of freshly prepared Aβ(1–42) and Aβ(1–38) reveal particles primarily with < 2 nm heights (Fig. 1B), which are generally associated with monomeric species^23^. Though the average volume values for the Aβ particles will be larger due to the AFM tip convolution effects, the general comparison between Aβ(1–38) and Aβ(1–42) shows that Aβ(1–38) has a larger particle volume (Fig. 1E), suggesting a different morphology or modest initial aggregation when compared to Aβ(1–42) particle morphology. With time, Aβ(1–42) progresses through to amyloid fibrils. while Aβ(1–42) co-incubated with Aβ(1–38) shows a dynamic profile that also begins relatively homogenously small and progresses (at 24 h) through a heterogeneous mix of heights and volumes (*e.g.* with two peaks at 2 and 6 nm in height and a shallow, but broad, volume peak at 50,000 nm^3^) (Fig. 1C,F), to return to a more homogenous mixture of particles that remain smaller and shorter after 48 h incubation (Fig. 1D,G).

**Figure 2:**
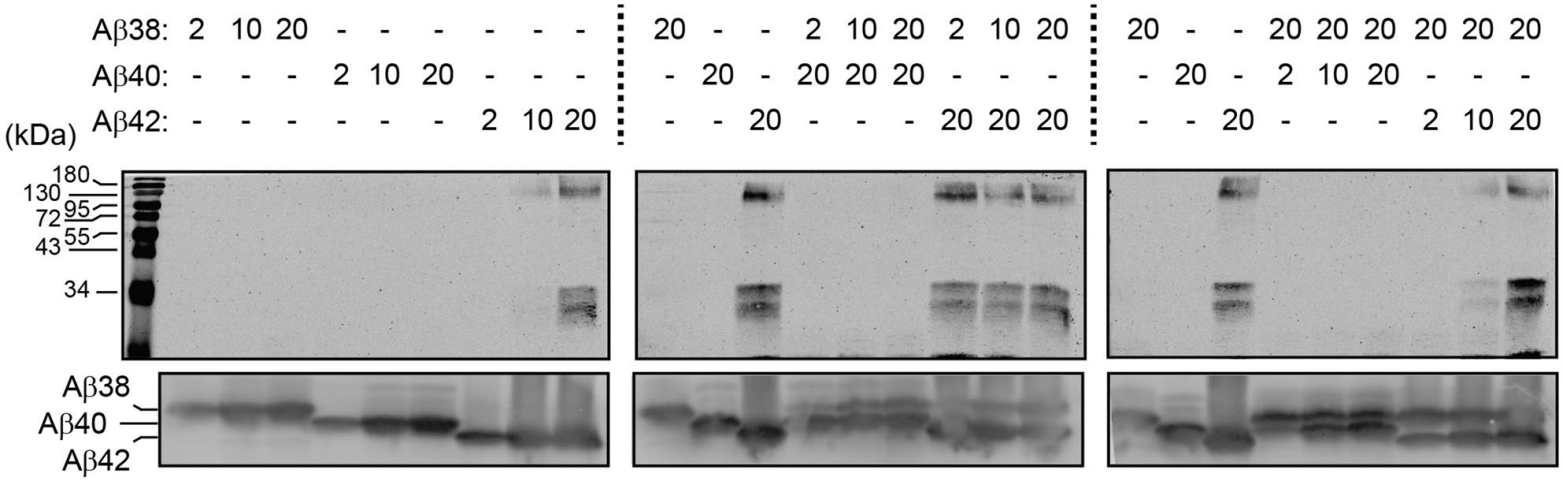
Aβ(1–38) interferes with Aβ(1–42) high molecular weight complexes on SDS-PAGE. Different concentrations (in µM) of Aβ(1–38), Aβ(1–40), or Aβ(1–42), either alone or as a mixture were incubated for 24 h and then resolved by gel electrophoresis. A protein ladder is shown in the first lane. Aβ high molecular weight complexes were visualized by standard 15% SDS-PAGE (*top panels*), while the different starting amounts of Aβ peptides themselves were visualized using an 8 M UREA / 12% SDS-PAGE system (*bottom blot*). Note that on urea gels, Aβ peptides migrate according to their hydrophobicity and, hence, Aβ(1–38) migrates slower (higher) than Aβ(1–40) or Aβ(1–42).

For our Western blotting experiments, we used increasing concentrations (2, 10, and 20 µM) to monitor potential aggregation of the various peptides. First, our blots confirm that HFIP-treated Aβ(1–38), Aβ(1–40), and Aβ(1–42) preparations contain significant amounts of monomeric species (*bottom panels*, Fig. 2), as expected^23,24^. In isolation, there are no detectable aggregates in the Aβ(1–38) or Aβ(1–40) solutions, even at concentrations of 20 µM (*top left panel*, Fig. 2). In contrast, the 10 and 20 µM solutions of Aβ(1–42) provide evidence of significant high molecular weight (HMW) aggregates. Co-incubation with increasing concentrations of Aβ(1–38) progressively lessens the amount of HMW aggregate associated with 20 µM Aβ(1–42) (*top middle and right panels*, Fig. 2). Mixtures of Aβ(1–38) and Aβ(1–40) do not present any evidence of HMW complexes. These observations confirm that Aβ(1–38) can interfere with Aβ(1–42) aggregation and, as importantly, suggest that although Aβ(1–38) and Aβ(1–42) aggregates are detected by AFM (see Fig. 1), these aggregates have profoundly different physicochemical properties, with, for example, Aβ(1–42) aggregates being stable on SDS-PAGE, whereas Aβ(1–38) aggregates are not.

**Figure 3:**
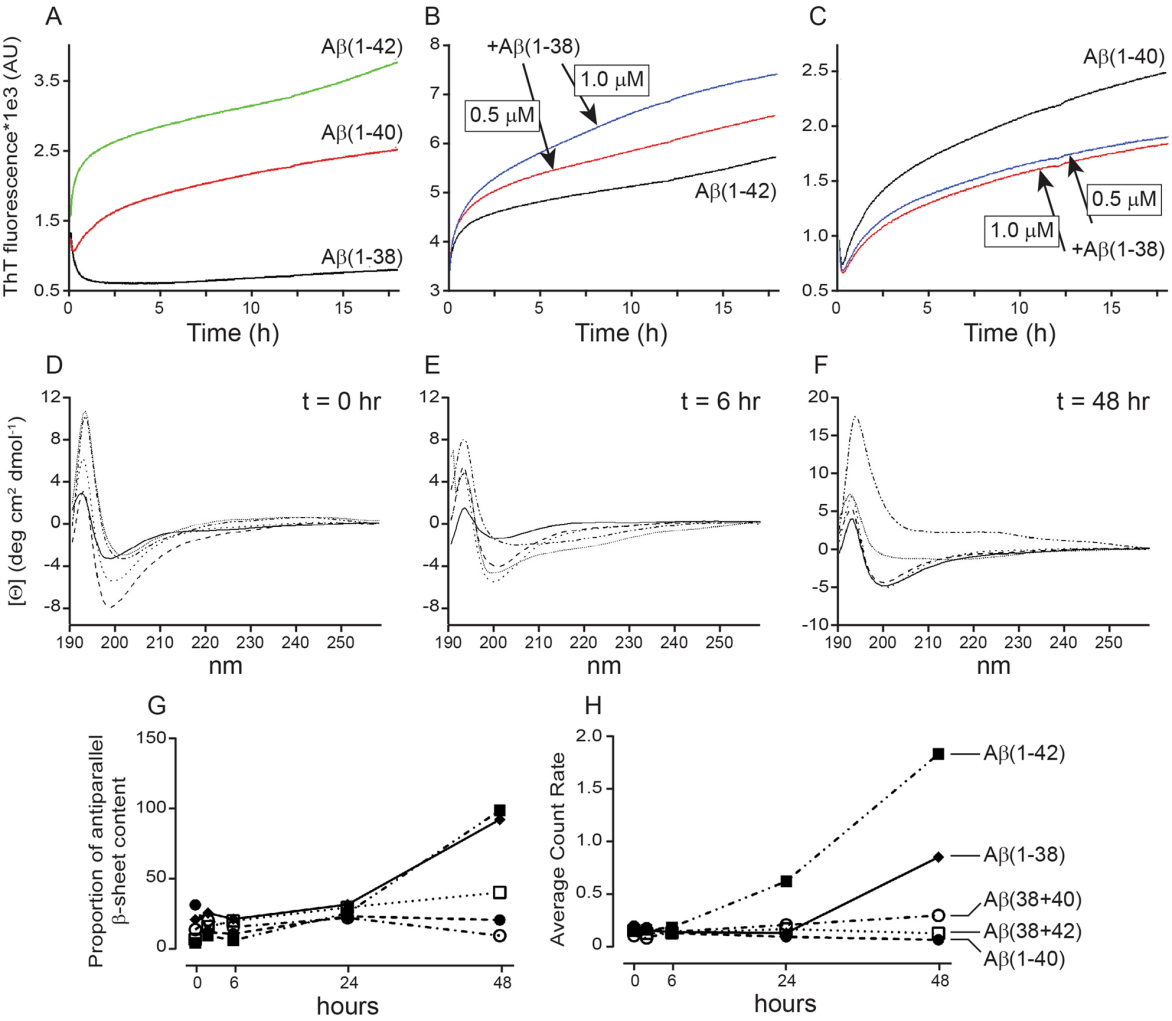
Aβ(1–38) reduces the β-sheet content and aggregation potential of Aβ(1–42). (**A**) ThT binding to Aβ peptides incubated in isolation or as a mixture. 20 µM of (**B**) Aβ(1–42) or (**C**) Aβ(1–40) was co-incubated with either 0.5 or 1 µM of Aβ(1–38) and monitored for ThT fluorescence over time. (**D–G**) CD spectroscopy reveals that the secondary structures of 20 µM Aβ(1–38), Aβ(1–40), and Aβ(1–42) can alter with time (*e.g.* 0, 6, and 48 h) and with co-incubation with 1 µM of Aβ(1–38). (**H**) Analysis of the oligomer size distributions of Aβ peptides (20 µM) by DLS reveals similar patterns over time to those captured by CD analysis. The line style in panel **H** also applies to the CD data in panels **D–G**. Data represent averages of three separate determinations.

There is a time-dependent increase in ThT fluorescence for Aβ(1–40) and Aβ(1–42) (Fig. 3A). While Aβ(1–38) in isolation causes an anomalous initial decrease in ThT fluorescence, this stabilizes over time. In a mixture, Aβ(1–38) exerts distinct effects on the other peptides, *e.g.* increasing ThT fluorescence with Aβ(1–42) (Fig. 3B), but decreasing ThT fluorescence with Aβ(1–40) (Fig. 3C). These data suggest differences in β-sheet content depending on whether the peptides are incubated in isolation versus in a mixture.

In isolation, Aβ(1–38) and Aβ(1–42) show a strong negative peak around 200 nm in the far-CD spectrum indicating an initial disordered state^25,26^, but with time the canonical anti-parallel β-sheet content emerges (Fig. 3D-G). A 20:1 Aβ(1–42):Aβ(1–38) solution has much less β-sheet growth, whereas a 20:1 Aβ(1–40):Aβ(1–38) solution appears to show evidence of some β-sheet growth that was not observed when Aβ(1–40) was incubated in isolation. These observations appear to contrast with our ThT binding data (*above*), but appear to align with our DLS measurements (Fig. 3H). Indeed, the scattering intensity, *e.g.* particle size, is lower over time in the Aβ(1–42):Aβ(1–38) mixture when compared to either peptide alone, whereas Aβ(1–40) particles in isolation are far smaller and tend to be modestly larger when co-incubated with Aβ(1–38) (Fig. 3H).

These cumulative biophysical data confirm that the individual peptides do demonstrate varying degrees of aggregation potential, but that co-incubation with Aβ(1–38) can trigger dramatically different aggregation behavior in Aβ(1–42) and Aβ(1–40). This further suggests that conclusions drawn from studies of Aβ peptides in isolation, while meaningful to understanding the behaviour of that particular peptide, likely cannot be generalized to the peptide’s behaviour in more complex biological mixtures. We chose to examine whether these biophysical trends extended to functional paradigms.

### Functional assays

**Figure 4:**
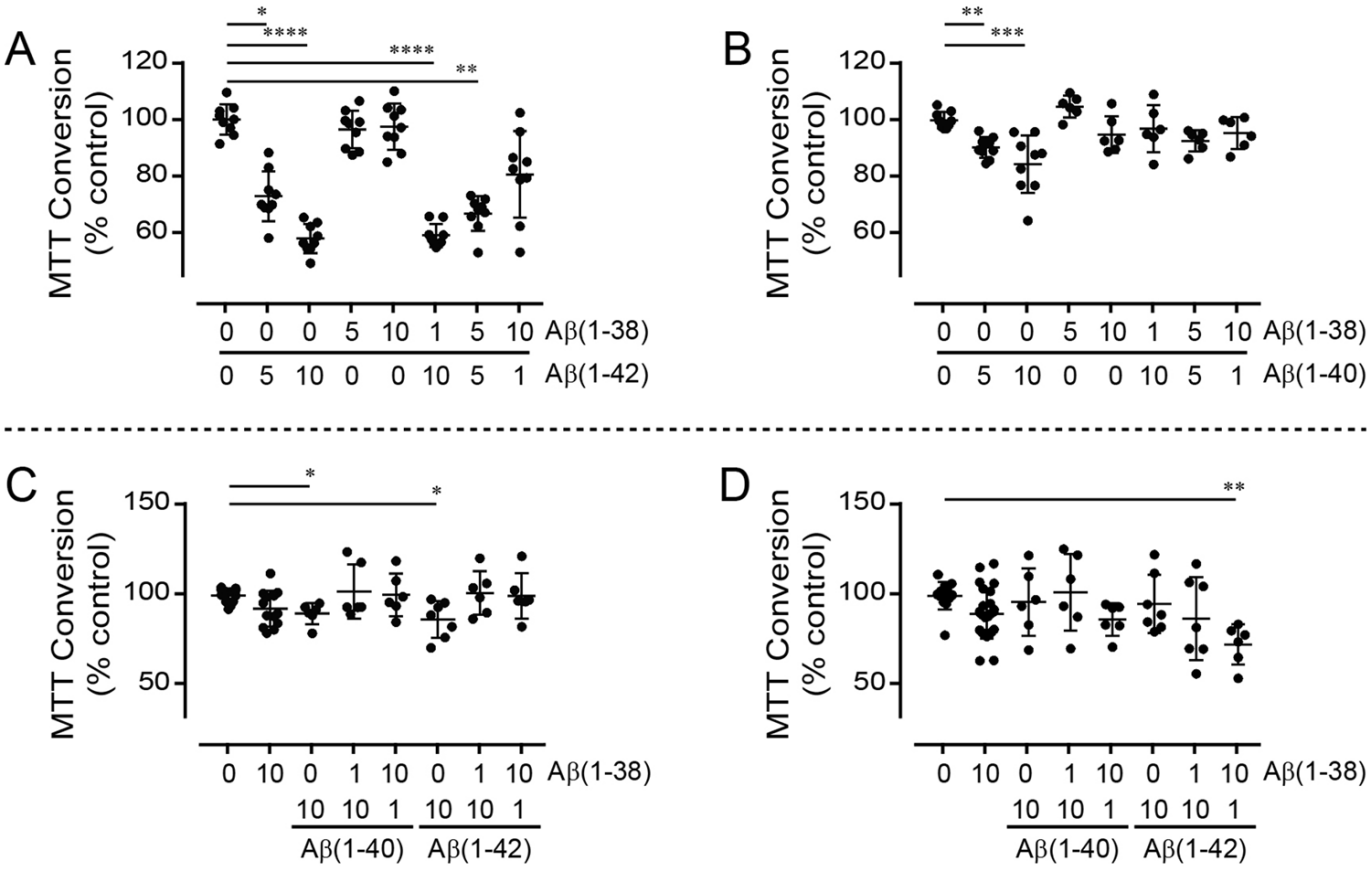
Mitochondrial metabolism (MTT conversion) used as a reflection of cell health in Aβ-treated murine hippocampal neuronal cells and human fibroblast cultures. The HT-22 cell cultures (n = 6–9) were treated (24 h) with concentrations of (**A**) Aβ(1–38) and Aβ(1–42) or (**B**) Aβ(1–38) and Aβ(1–40), as indicated along the X-axis (labels are in µM). (**C**) *APOE* ε4-positive and (**D**) *APOE* ε4-negative fibroblast cultures (n = 6–12) were treated (24 h) with combinations of either Aβ(1–38), Aβ(1–40), and Aβ(1–42) as indicated along the X-axis (labels are in µM). *: *P* < 0.05; **: *P* < 0.01; ***: *P* < 0.001; ****: *P* < 0.0001 *vs.* ‘0/0′-treated control cultures.

Freshly prepared Aβ(1–42) decreases mitochondrial respiration (*e.g.* MTT conversion) in a concentration-dependent manner [*P* < 0.0001], whereas Aβ(1–38) on its own has no effect (Fig. 4A). Co-treatment with Aβ(1–42) and differing ratios of Aβ(1–38) does not produce any effect that cannot be simply attributed to a titration of the Aβ(1–42) effect. Interestingly, a combination of Aβ(1–38) and subequimolar concentration of Aβ(1–42) appears to reduce mitochondrial respiration compared to Aβ(1–38) alone, although the effect does not reach statistical significance (Fig. 4A). In contrast, the effect of Aβ(1–40) [*P* < 0.0001) is completely inhibited by co-treatment with Aβ(1–38) in HT-22 cells (Fig. 4B).

To determine whether *APOE* ε4, a risk allele for late-onset AD in women (discussed in^27^), might influence outcomes, we tested the peptides in two human fibroblast cell lines from female donors that differed in their *APOE* ε4 status. MTT conversion tends to be marginally affected in the *APOE* ε4/ε4 fibroblast cell line by Aβ(1–40) (*P* = 0.0629) and Aβ(1–42) (*P* = 0.0814) (Fig. 4C). *Post-hoc* analysis shows that Aβ(1–38) exerts no effect on its own in this cell line, but reverses the modest effects exerted by both Aβ(1–40) and Aβ(1–42). In contrast, the peptides do not exert any effect in the *APOE* ε2/ε3 fibroblast cell line (Fig. 4D), although significance (*P* < 0.01) across the treatment groups is detected; *post-hoc* analysis reveals that a subequimolar concentration of Aβ(1–42) exacerbates the effect of Aβ(1–38), while a similar subequimolar concentration of Aβ(1–40) does not (Fig. 4D). This intriguing observation warrants further investigation.

Since Aβ peptides, and Aβ(1–42) in particular, are known to target the plasma membrane and associated functions, we chose to examine how our Aβ peptide combinations might influence electrophysiological processes.

**Figure 5:**
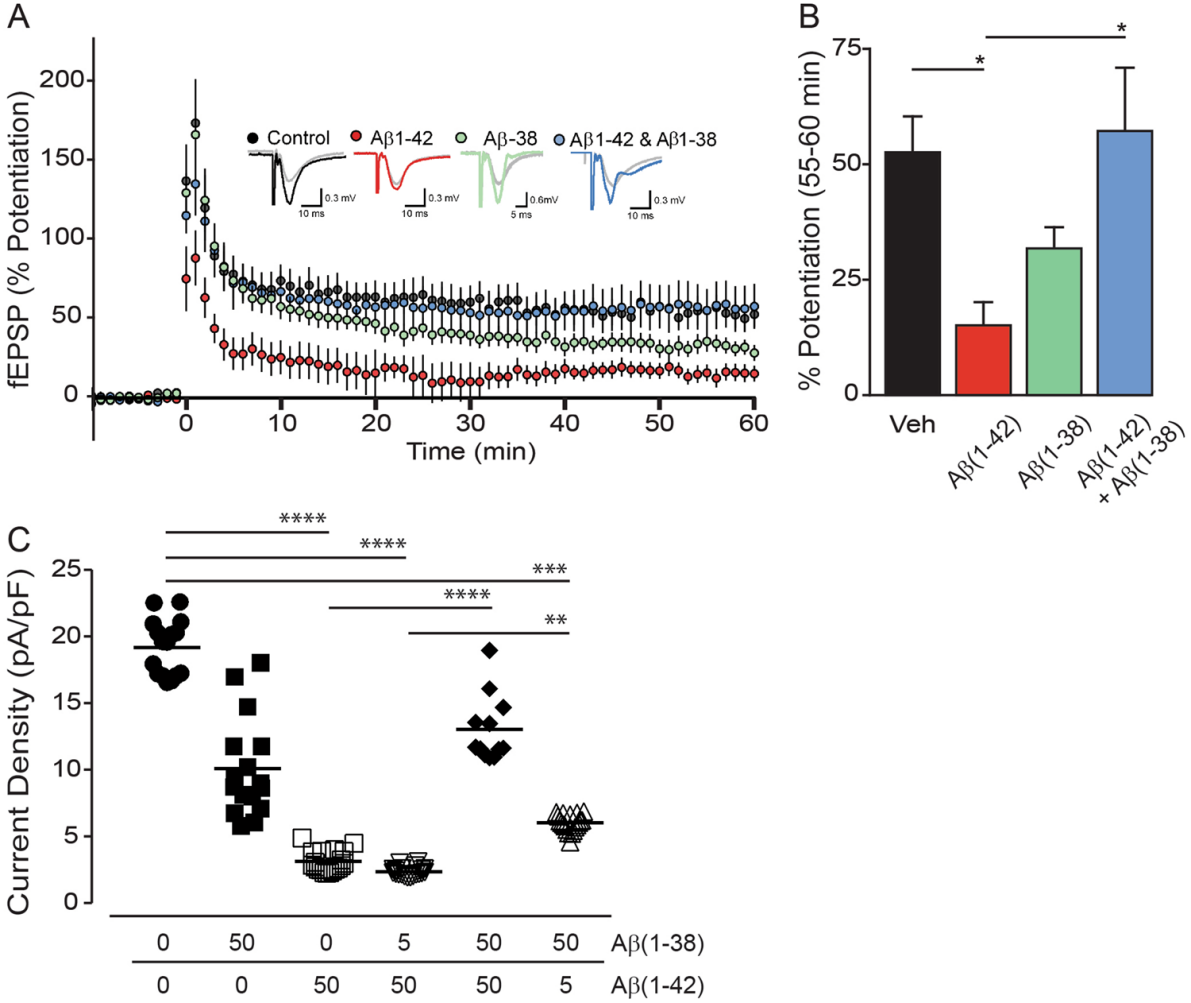
Aβ(1–38) rescues the deficit caused by application of Aβ(1–42) in electrophysiological paradigms. (**A**) Long-term potentiation (LTP) graph showing the percent potentiation (mean ± sem) following theta burst LTP in control (black; *n* = 10), Aβ(1–42) (red; *n* = 8), Aβ(1–38) (green; *n* = 6), and Aβ(1–42) + Aβ(1–38) (blue; *n* = 7) conditions (all peptides at 500 nM). Theta burst stimulation was applied at time = 0 min. Representative fEPSP responses obtained during the baseline (grey traces) and 55–60 min following LTP induction (black, red, blue and green traces) are shown for each condition. (**B**) Bar graph showing the percent potentiation (mean ± sem) 55–60 min post-LTP induction. *: *P* < 0.05. (**C**) Average current densities (± sem) from whole cell voltage-clamp recordings in primary hippocampal neurons exposed to Aβ(1–38) and Aβ(1–42) (in µM, 24 h). Cells were held at -60 mV and currents were elicited by voltage ramps, *e.g.*, step depolarized between -80 and + 100 mV (in 20 mV increments). Current density was measured at 0 and 20 mV, 100 ms after the voltage step. Currents were divided by cell capacitance and reported as current densities (pA/pF). **: *P* < 0.01; ***: *P* < 0.001; ****: *P* < 0.0001 between indicated groups.

In control hippocampal slices, theta burst stimulation results in robust LTP, as measured by the percent increase in fEPSP slope 55–60 min post-LTP induction (52.90 ± 7.97%, n = 10) (Fig. 5A,B). As expected, 500 nM Aβ(1–42) inhibits LTP (15.04 ± 5.35%, n = 8; *P* < 0.05). When 500 nM Aβ(1–38) is applied alone, the mean percent potentiation is reduced, but not significantly different from controls (31.57 ± 4.96%, n = 6), corroborating previous observations, *e.g.*^21^. Remarkably, when Aβ(1–38) is co-applied with Aβ(1–42) (both at 500 nM), the resultant LTP evoked by theta burst stimulation is completely restored to control levels (57.03 ± 14.38%, n = 7).

Patch-clamping reveals that hippocampal neuron current densities, *e.g.* currents normalized to the cell capacitance and an index of membrane permeability, are highest (18.97 pA/pF ± 2.15) in non-Aβ exposed neurons (CTL) (Fig. 5C). Exposure to Aβ(1–38) causes a significant decrease in current density (9.21 pA/pF ± 3.85, *P* = 0.007) and exposure to Aβ(1–42) leads to an even lower current density (3.10 pA/pF ± 1.91, *P* = 0.01), and yet, as with the LTP paradigm, co-treatment with Aβ(1–38) rescues the effect of Aβ(1–42). Interestingly, the decrease in current density observed for Aβ(1–38) and a subequimolar concentration of Aβ(1–42) is more in the range of current density measured when neurons are exposed to Aβ(1–42) alone. As Aβ peptides decrease the current, the membrane resistance is increased and the membrane potential that is measured is -80 mV (reflecting the closure of K^+^ channels).

These two electrophysiological paradigms confirm that Aβ(1–38) has the capacity to negatively regulate Aβ(1–42)-mediated deficits at the synapse and the cell membrane. We used the *C. elegans* worm to test whether any ‘protection’ afforded by Aβ(1–38) might extend to an in vivo context.

**Figure 6:**
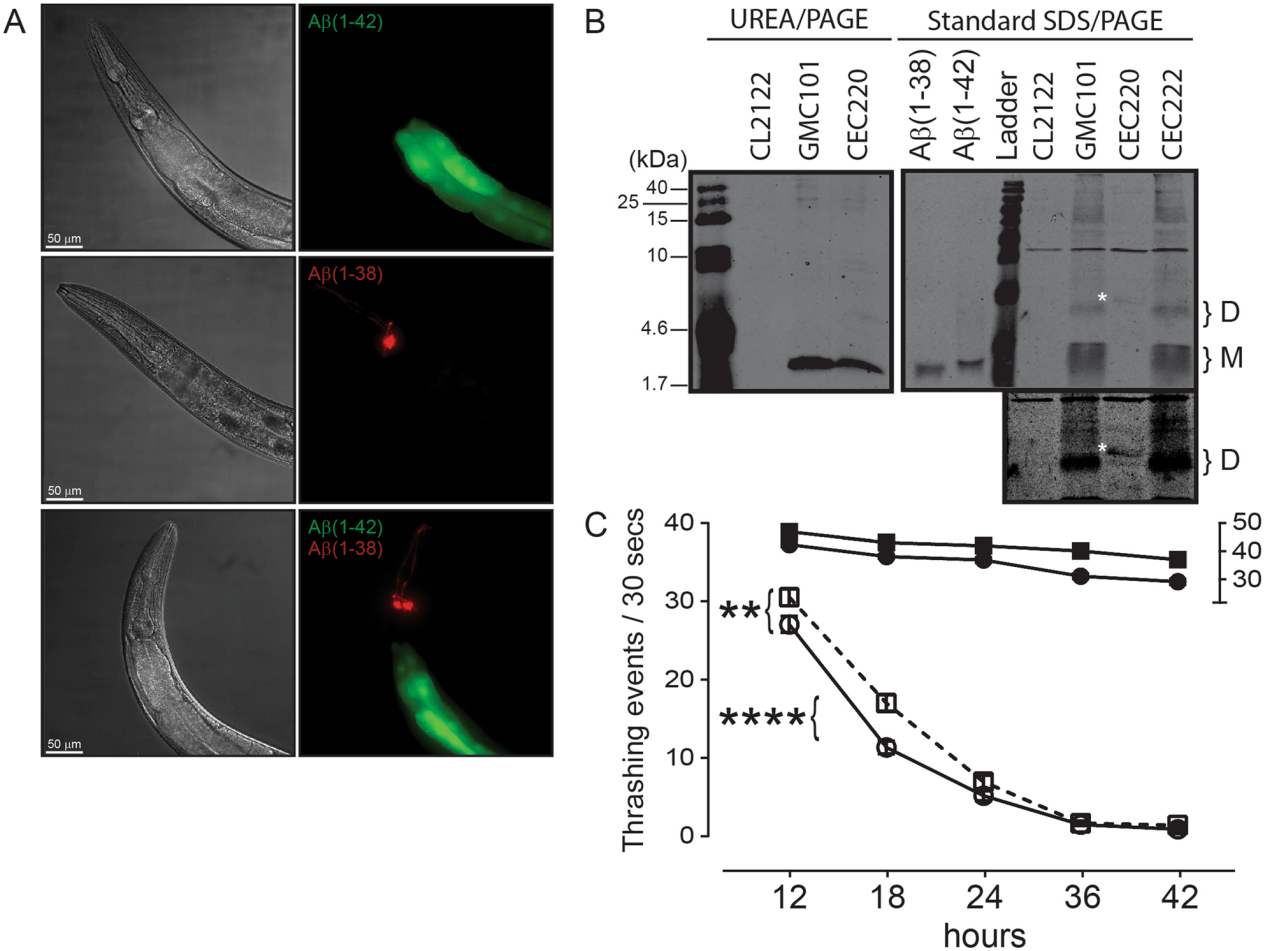
Aβ(1–38) can partially suppress Aβ(1–42)-dependent muscle deterioration in *C. elegans*. (**A**) Representative images of the anterior region of worms overexpressing untagged Aβ peptides in body wall muscle. DIC images (*left panels*) and live fluorescence (*right panels*) represent: Aβ(1–42) (GMC101 strain; transgene marked by GFP (green) in the intestine), Aβ(1–38) (CEC220 strain; transgene marked by DsRed (red) in head neurons), and a double transgenic animal (CEC222 strain) expressing both Aβ(1–42) and Aβ1(-38) peptides (both green and red markers present). Scale bar, 50 μm; magnification, 50X. (**B**) Representative immunoblot of UREA/SDS-PAGE resolved extracts showing the expression of Aβ(1–42) in the GMC101 strain and Aβ(1–38) in the CEC220 strain. The protein ladder (in kDa) is indicated on the left. Extracts were also resolved by standard 15% SDS-PAGE and reveal putative monomers (M) and dimers (D). A band in the CEC220 extract (identified with white asterisk ‘*’) likely represents a modified Aβ(1–38) dimer, which is seen more clearly in a longer exposure (*lower panel*). (**C**) Time-dependent changes in the thrashing rates in CL2122 (control) worms (filled circle) and worms expressing Aβ(1–38) (filled square) (relative to the ‘30–50’ segment on the right Y-axis) as well as in the Aβ(1–42)-expressing GMC101 strain (○), and worms co-expressing Aβ(1–38) and Aβ(1–42) phenotype (□). Two-way ANOVA shows that all groups were significantly different from their respective control groups (n = 60–90; mean ± sem). **: *P* = 0.01; ****: *P* = 0.0001 between indicated groups.

The *C. elegans* GMC1010 strain expresses full length Aβ(1–42) in body wall muscle cells and exhibits a paralysis phenotype^28^. We used ‘thrashing rate’ as a proxy for compromised muscle function due to Aβ accumulation. The CL2122 strain, which expresses GFP in the intestine, but produces no Aβ peptide, was used as a control. The presence of muscle-specific Aβ(1–42) and/or Aβ(1–38) in these worms is inferred with detection of gut GFP and neuronal DsRed, respectively (Fig. 6A). Western blotting confirms the expression of Aβ(1–42) in the GCM101 strain and Aβ(1–38) in the CEC220 strain (Fig. 6B). However, both peptides show a similar mobility on Urea/PAGE, which suggests an increase in hydrophobicity of the Aβ(1–38) species^29^ in this worm, presumably through some post-translational modification. Samples resolved on standard 15% SDS-PAGE (Fig. 6B) reveal that a putative Aβ(1–38) dimer migrates *higher* than an Aβ(1–42) dimer, confirming a modified, *e.g.* heavier, Aβ(1–38) species.

At 12-h post-L4 larval stage, the thrashing rate in synchronized populations of Aβ(1–42)-expressing worms (GMC101) was reduced (*P* < 0.0001) as compared to the control CL2122 strain, but not compared to Aβ(1–38)-expressing worms (CEC220) (Fig. 6C). The motor defect observed in GMC101 worms is consistent with a previous report^28^. At 18 h, the thrashing rates is significantly higher in CEC222 worms, which express both Aβ(1–38) and Aβ(1–42) peptides, when compared to worms that express Aβ(1–42) alone [two-way ANOVA, Interaction *P* = 0.0004]. At 24 h, this difference is less pronounced, but remains significant, and by 36 h any difference is lost (Fig. 6C).

The *C. elegans* data suggest that any potential benefit attributable to Aβ(1–38) might be masked if Aβ(1–42) accumulates within the same tissue. The AD brain tends to accumulate numerous Aβ species and we recently demonstrated that levels of insoluble (guanidine-extractable) Aβ peptides differed in a region- and sex-dependent manner in brain samples from autopsy-confirmed cases of AD^27^. We chose to examine how levels of *soluble* Aβ peptides relate to each other in these same samples.

### Clinical autopsy samples

**Figure 7:**
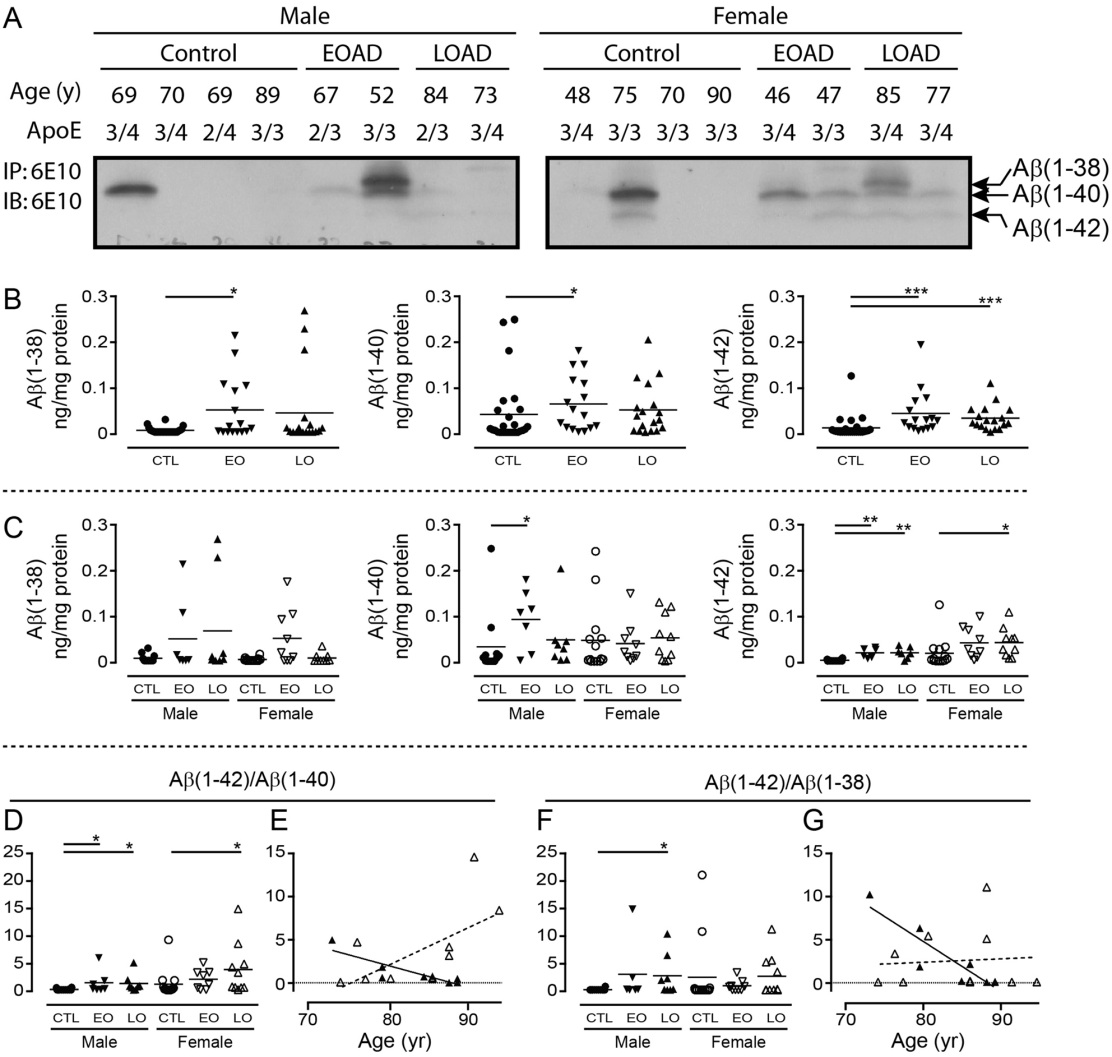
Levels of Aβ peptides isolated from RIPA-soluble human cortical extracts. (**A**) Representative gels showing resolved Aβ peptides isolated by 6E10-immunoprecipitation and used for densitometric analyses. Bands corresponding to Aβ(1–38), Aβ(1–40), and Aβ(1–42) are identified (based on a set of synthetic standards run separately; *not shown*). Samples represent male and female donors diagnosed with Early-Onset AD (EOAD) or Late-Onset AD (LOAD), or from age- and sex-matched controls. Respective age-at-death and *APOE* genotype are indicated. Levels of peptides (in ng *per* mg protein) were analyzed by (**B**) diagnosis or (**C**) stratified by sex and diagnosis. The data were expressed as (**D**) Aβ(1–42)/Aβ(1–40) or (**F**) Aβ(1–42)/Aβ(1–38) ratios. (**E**, **G**) The relation between these ratios and age of the donor at autopsy were examined by regression (Pearson’s) analysis. *: *P* < 0.05; **: *P* < 0.01; ***: *P* < 0.001 *vs.* corresponding controls (CTL). EO: Early-Onset AD; LO: Late-Onset AD. An example of a full-length blot is presented in Supplementary Fig. 1.

Early-onset (EO) and late-onset (LO) AD donor statistics are presented in Table 1. Western blotting of RIPA-extracts of cortical samples clearly reveals bands corresponding to Aβ(1–38), Aβ(1–40), and Aβ(1–42) (Fig. 7A). Levels of Aβ(1–38) [*P* = 0.0207] and Aβ(1–40) [*P* = 0.0234] are higher in EOAD samples, and levels of Aβ(1–42) [*P* < 0.0001] is higher in both EOAD and LOAD samples versus levels in neurologically normal controls (Fig. 7B), with contributions from both sexes (Fig. 7C). The Aβ(1–42)/Aβ(1–40) ratio increases in the LOAD cortex [*P* < 0.0001] and correlates negatively with age-at-death in males [*P* = 0.0053, r = 0.8668] and tends to correlate with age-at-death in females [*P* = 0.0568, r = 0.6181] (Fig. 7D,E). A significantly higher Aβ(1–42)/Aβ(1–38) ratio [*P* = 0.0087] in cortex is driven exclusively by male LOAD samples and is also negatively correlated with the age-at-death [*P* = 0.0024; r = 0.8991]. There is no such correlation in female LOAD samples [*P* = 0.8416] (Fig. 7F,G).

**Table 1:** Basic donor parameters.

	Control	Early-Onset AD	Late-Onset AD
Sex	12 M/14 F	7 M/9 F	8 M/10 F
Age (years)			
M	70.7 ± 9.85	63.1 ± 5.61	82.9 ± 5.38*
F	70.8 ± 14.8	54.2 ± 7.16*	83.4 ± 6.35
PMI (hours)			
M	16.7 ± 8.50	25.2 ± 10.6	22.7 ± 8.54
F	21.7 ± 12.7	18.5 ± 9.87	20.4 ± 7.98
Brain weight (grams)			
M	1275 ± 155.1	1157 ± 139.2	1126 ± 98.9*
F	1191 ± 108.6	879.4 ± 143.4***	992.0 ± 94.9*

The brain samples analyzed for soluble Aβ peptides were obtained from neurologically normal cases (Control) and from histopathologically confirmed cases of Early-Onset or Late-Onset Alzheimer disease (AD). M, male; F, female; years (time-at-death; age at autopsy); PMI, post-mortem interval; *P < 0.05; ***P < 0.001; *vs.* control donors (mean ± sem).

**Figure 8:**
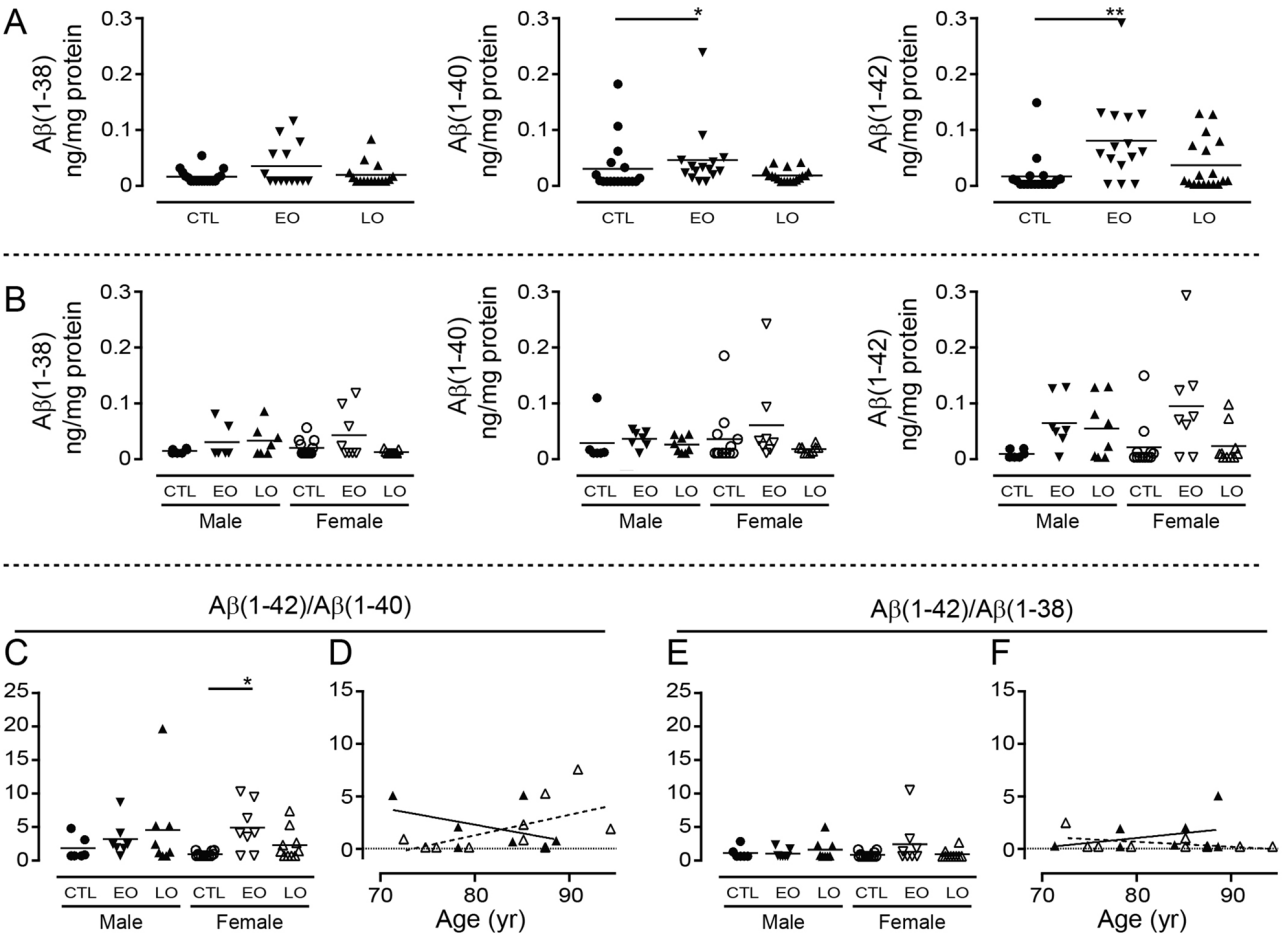
Levels of Aβ peptides isolated from RIPA-soluble human hippocampal extracts. Densitometric analyses were performed on the hippocampal samples from the same donors as described in Fig. 7. Levels of peptides were analyzed by (**A**) diagnosis or (**B**) stratified by sex and diagnosis. The data were expressed as (**C**) Aβ(1–42)/Aβ(1–40) or (**E**) Aβ(1–42)/Aβ(1–38) ratios and (**D**, **F**) the relations between these ratios and age of the donor at autopsy were examined by regression analysis. *: *P* < 0.05; **: *P* < 0.01 *vs.* corresponding controls (CTL). EO: Early-Onset AD; LO: Late-Onset AD.

In the corresponding hippocampal samples only Aβ(1–40) [*P* = 0.03] and Aβ(1–42) [*P* = 0.0081] levels are higher (and driven by EOAD samples); any significance is lost when the data are stratified by sex (Fig. 8A,B). The Aβ(1–42)/Aβ(1–40) ratio tends to increase [*P* = 0.080] (primarily in female EOAD samples) (Fig. 8C), while the Aβ(1–42)/Aβ(1–38) ratio is unchanged from that of controls [*P* = 0.5550] (Fig. 8E]. Neither Aβ(1–42)/Aβ(1–40) [male: *P* = 0.3071; female: *P* = 0.0902] or Aβ(1–42)/Aβ(1–38) [male: *P* = 0.4194; female: *P* = 0.1635] correlates with age-at-death (Fig. 8D,F).

**Figure 9:**
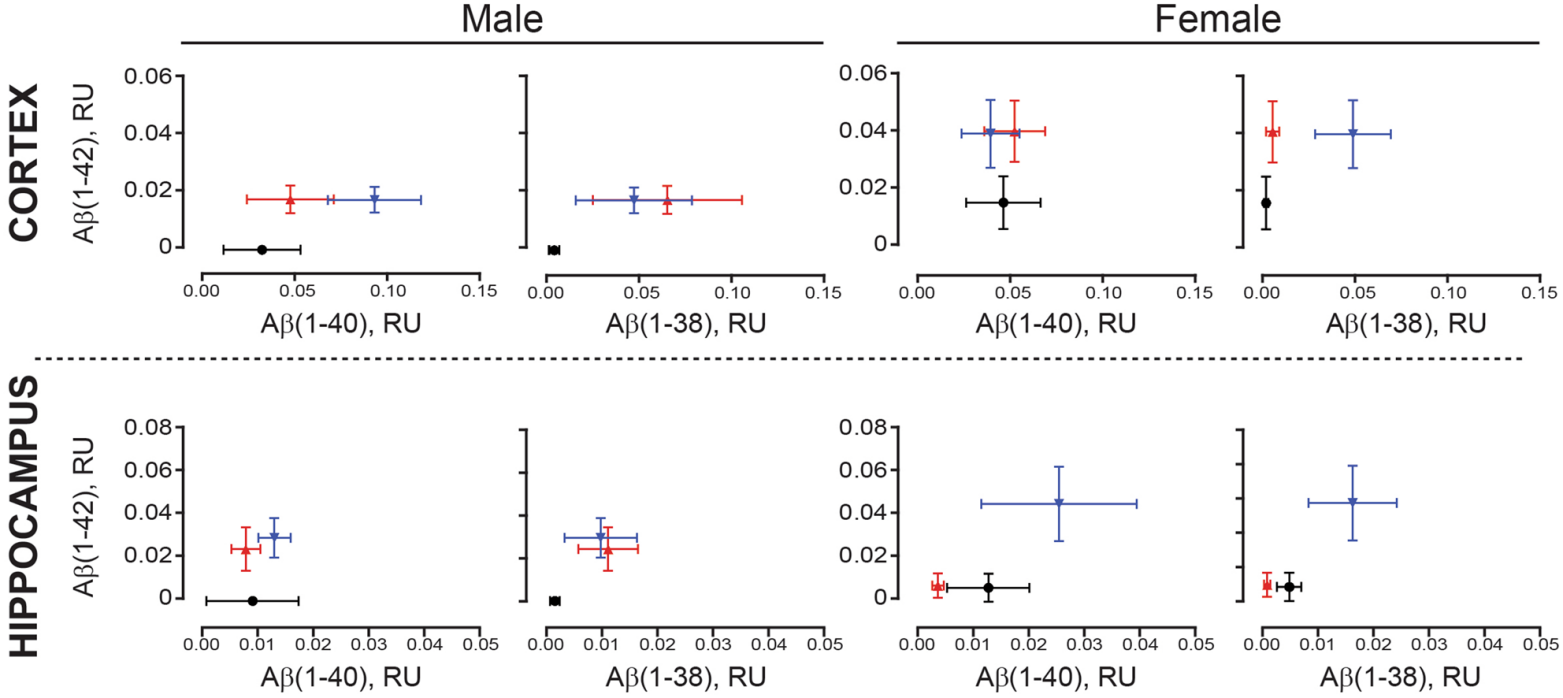
Aβ levels from cortical and hippocampal extracts were visualized as gnu plots. Cortical (*top panels*) and corresponding hippocampal (*bottom panels*) levels of Aβ(1–42), Aβ(1–40), and Aβ(1–38) were compared using gnu plots (mean ± sem). Control values are shown in black (filled circle), EOAD values are shown in blue (filled inverted triangle); LOAD values are shown in red (filled triangle).

For ease of interpretation, the relative proportions of Aβ(1–42) to either Aβ(1–38) or Aβ(1–40) are presented as gnu plots (Fig. 9). In both regions, the relative abundance of Aβ(1–38), Aβ(1–40), and Aβ(1–42) is greater in EOAD samples. There is generally more of the three peptides in males with LOAD in both regions. In contrast, while all three peptides are detectably higher in female cortical EOAD (*vs*. control) samples, levels in the corresponding hippocampal LOAD samples are unchanged from those in female controls (Fig. 9).

Parenthetically, it is known that protein accounts for 10% of brain wet weight^30^ and that brain density (*e.g.* g per cm^3^) is approximately ‘1’^31^. Using these factors, we were able to convert our data, expressed in ‘ng *per* mg protein’, to ‘ng *per* gram wet weight tissue’ and our rough estimates for the cortical samples are: 0.360 [for Aβ(1–38)], 3.998 [for Aβ(1–40)], and 0.968 [for Aβ(1–42)] for a total soluble Aβ(38 + 40 + 42) peptide of 5.326 ng *per* g tissue for controls. Using the same calculation, we obtained (in ‘ng *per* g tissue’) 4.895 [for Aβ(1–38)], 6.308 [for Aβ(1–40)], and 4.150 [for Aβ(1–42)] for a total of 15.353 soluble Aβ(38 + 40 + 42) peptide in early-onset AD samples and 4.267 [for Aβ(1–38)], 5.042 [for Aβ(1–40)], and 3.096 [for Aβ(1–42)] for a total of 12.405 soluble Aβ(38 + 40 + 42) peptide in late-onset AD samples. These estimates are remarkably in-line with estimates from a PET imaging study that estimated Aβ levels in the TBS (soluble) fraction of controls at 9.487 ng *per* cm^3^ (gram) and those of AD patients at 28.169 ng *per* cm^3^^32^.

Finally, so as to determine whether *APOE* ε*4* status might be influencing any of these autopsy-derived data, we re-analyzed the data by stratifying for *APOE* ε*4* status and sex (independent of diagnosis as samples sizes were too small). In the cortical samples, any significant effects (*data not shown*) were limited to: an increase in Aβ(1–42) [*P* = 0.0068], with contributions by both males [*P* = 0.0428] and females [*P* = 0.0361]; an increase in Aβ(1–42)/Aβ(1–40) [*P* = 0.0062], with contributions primarily from females [male: *P* = 0.0962; female: *P* = 0.0084]; and an increase in the Aβ(1–42)/Aβ(1–38) ratio [*P* = 0.0144] driven my males [*P* = 0.0283], but not females [*P* = 0.2079]. There were no effects of *APOE* ε*4* status on soluble peptide levels in the hippocampal samples.

Our observations indicate region- and sex-dependent proportions of these Aβ peptides in the AD brain. Up to this point, we have examined the effect of peptides one-on-one. We chose to determine what might transpire should different proportions of all three of the Aβ peptides be allowed to interact in a mixture. To do so, while still keeping the experimental design manageable, we monitored peptide-peptide interactions in real-time using surface plasmon resonance.

### Surface plasmon resonance (SPR)

**Figure 10:**
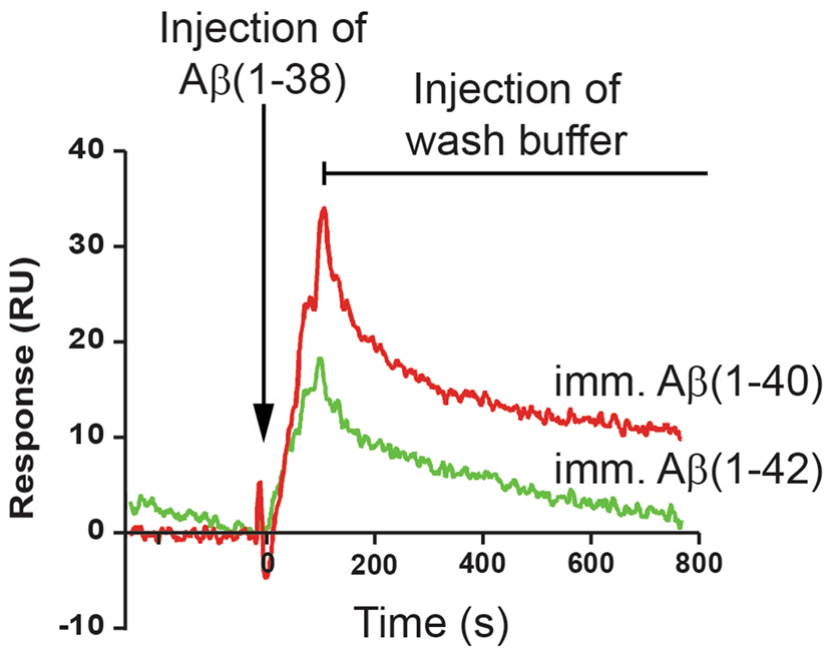
Surface plasmon resonance reveals Aβ(1–38) can interact directly with Aβ(1–42) and Aβ(1–40). A biosensor chip was prepared by immobilizing (imm.) freshly prepared Aβ(1–40) or Aβ(1–42) peptides on to flow cell surfaces. A 1200 nM solution of the analyte, *e.g.* Aβ(1–38), was injected (5 µL/min) for 120 s over the surfaces, and then replaced with wash buffer, thus yielding association and dissociation phases, respectively. The binding of Aβ(1–38) with Aβ(1–40) is depicted by the red sensorgram, while the binding with Aβ(1–42) is depicted by the green sensorgram.

**Figure 11:**
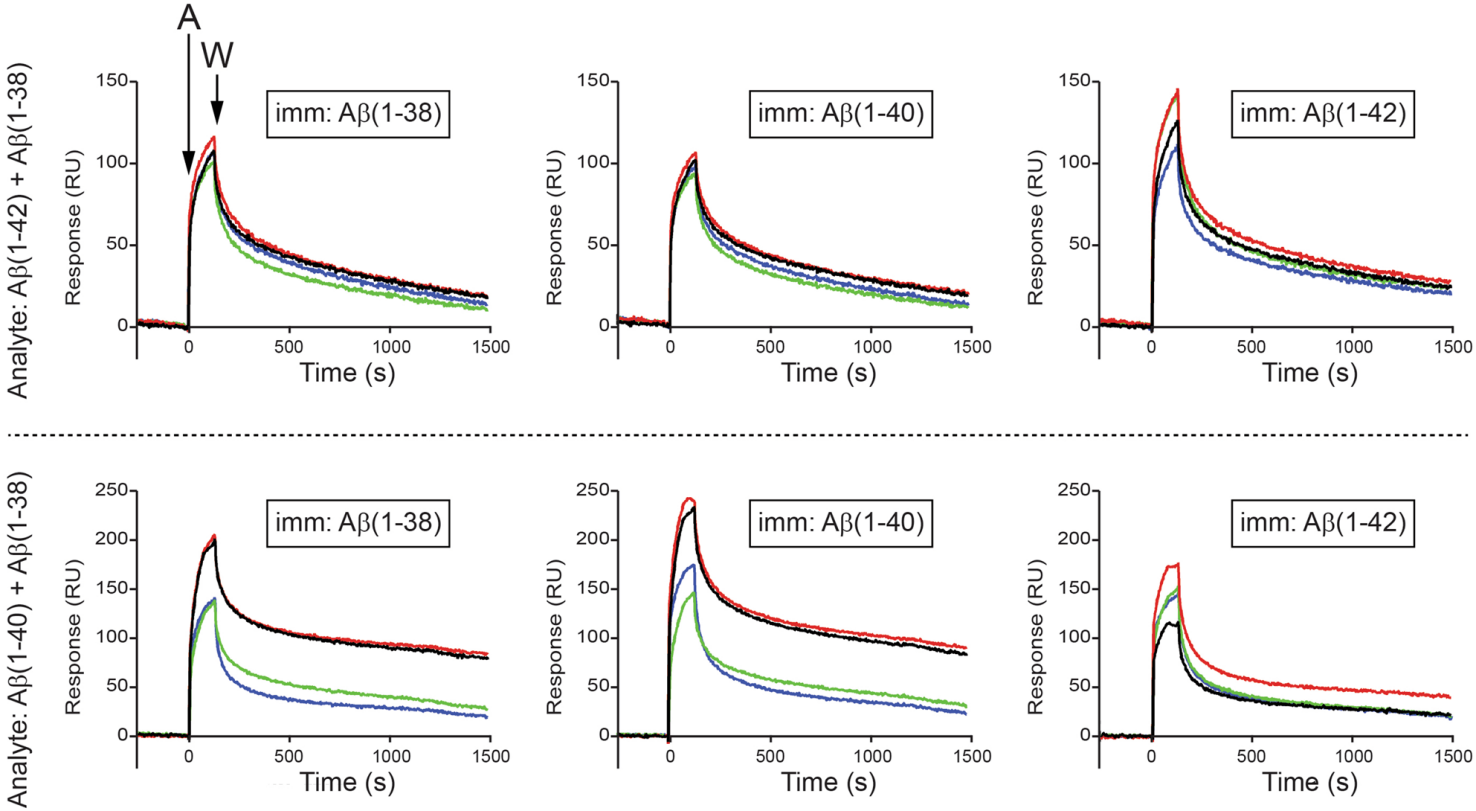
Surface plasmon resonance reveals complex interactions between Aβ(1–42), Aβ(1–40), and Aβ(1–38). Freshly prepared Aβ(1–38), Aβ(1–40), or Aβ(1–42) peptides were immobilized (imm.) on to biosensor chips. Test (analyte: ‘A’) solutions injected (5 µL/min for 120 s) over the surfaces contained 1200 nM of either (*left panels*) Aβ(1–42) or (*right panels*) Aβ(1–40) either alone (black sensorgrams) or also containing 50 nM Aβ(1–38) (red sensorgrams); 100 nM Aβ(1–38) (green sensorgrams); or 200 nM Aβ(1–38) (blue sensorgrams). The dissociation phase of the binding was monitored by replacing the analyte solution with wash buffer (‘W’). Sensorgrams are representative of three or more experimental replicates.

Approximately 1600 relative units (RUs) of either Aβ(1–40) or Aβ(1–42) were immobilized on a biosensor chip. First, we demonstrate that Aβ(1–38) binds more with Aβ(1–40) than with Aβ(1–42) (Fig. 10). Then, we demonstrate that Aβ(1–42) (injected at 1200 nM) recognizes all three immobilized peptides, *e.g.* Aβ(1–38), Aβ(1–40) and itself, and that addition of increasing concentrations (50–200 nM) of Aβ(1–38) to the Aβ(1–42) solution does not significantly alter the interaction between the injected Aβ(1–42) and any of the immobilized peptides (*top panels*, Fig. 11). Injected Aβ(1–40) also recognizes all three immobilized peptides and we observed a concentration-dependent inhibition by Aβ(1–38) of the interaction between the injected Aβ(1–40) and the immobilized Aβ(1–38) or itself (*bottom panels*, Fig. 11). In contrast, a low concentration of Aβ(1–38) significantly increases the interaction between the injected Aβ(1–40) and immobilized Aβ(1–42), and increasing the concentration of Aβ(1–38) in the injected solution diminishes this effect.

These observations confirm that the three peptides can interact in a complex mixture and that Aβ(1–38), under these circumstances, might interfere with Aβ(1–40) self-aggregation, but could promote the interaction between Aβ(1–40) and Aβ(1–42). This could be fundamental for our understanding of amyloid plaque formation in the AD brain.

## Discussion

Although there are countless reports regarding the behaviour of Aβ peptides in isolation, very little is known of their properties when incubated as complex mixtures or when studied immediately upon reconstitution of HFIP-treated stocks, when the peptides would have the lowest percentage possible of β-sheet structure^24^, which would more closely reflect the state immediately upon synthesis in vivo.

Our biophysical analyses confirm that these peptides exhibit significant differences in size distribution and time-dependent genesis of secondary structural elements^23,33^, with the properties of Aβ(1–38) lying between those of Aβ(1–40) and Aβ(1–42)^22^. We also demonstrate that co-incubation of Aβ(1–38) with Aβ(1–42) mitigates fibril length and aggregate size as well as overall β-sheet content of any interacting complex, and these differences in biophysical profiles generally align with differences in functional profiles.

Aβ(1–42) in the form of higher molecular weight aggregates^18,34^ as well as low molecular weight and toxic oligomers composed of dimers, trimers, and tetramers^33^ has been shown to inhibit LTP, by way of interactions with the phospholipids of the plasma membrane^35^ or with specific receptors, *e.g.* the insulin receptor^36^ or cholinergic receptors^37^. The Aβ peptides also directly alter conductance centered on calcium homeostasis, with the latter implicating specific calcium channels^38^, NMDA^39^ or AMPA^40^ receptors, or a role for channel formation by Aβ(1–42) itself^41,42^. Longer, soluble Aβ peptides, including Aβ(1–42), alter synaptic plasticity and impair hippocampal LTP^43,44^, while the shorter peptides, including Aβ(1–37/38/39/40), are less likely to elicit any overt effect on synaptic function^21^. Our current studies confirm a significant inhibition of LTP by soluble Aβ(1–42) and a modest (~ 20%), albeit not statistically significant, inhibition of LTP by Aβ(1–38); however co-treatment with the two peptides completely rescues the impaired LTP phenotype observed with Aβ(1–42) alone. Similarly, patch-clamping shows an Aβ(1–42)-dependent loss of current density in primary hippocampal neurons, which is reversed by an equimolar concentration of Aβ(1–38). Although the actual mechanism needs to be defined, our observed changes in current density suggest that Aβ(1–38) might be mitigating a disruption of membrane permeability that has been demonstrated elsewhere by changes in intracellular Ca^2+^ (also discussed above) or increased influx of dye (*e.g.* ethidium bromide) following treatment with Aβ(1–42)^45^. Another possibility might be that Aβ(1–38) is disrupting self-association of Aβ(1–42) into an ion-conducting channel or pore structure that is not evident with shorter peptides, *e.g.* Aβ(1–40)^42^, in such acute treatment paradigms. Our observations have significant implications for the influence of Aβ length variants on neuronal membrane integrity, synaptic plasticity, and memory formation.

In our HT-22 murine hippocampal cultures, subequimolar Aβ(1–38) fully rescues Aβ(1–40)-mediated mitochondrial dysfunction, but has no obvious effect on Aβ(1–42). This is in contrast to the report that Aβ(1–38) could trigger an Aβ(1–40)-mediated cytotoxicity, but could also protect against Aβ(1–42)-mediated toxicity in the SH-Sy5y neuroblastoma cell line^22^. Perhaps this discrepancy reflects the use of aged Aβ peptides in that study^22^. Indeed, soluble *versus* aged/aggregated Aβ peptide has been shown to elicit distinct phenotypes in SH-Sy5y cells^46^.

Our two human fibroblast cell lines, both from female donors, but differing in their *APOE* ε4 status (a risk for AD in women), also yielded intriguing results. This *APOE* ε4/ε4 fibroblast line is modestly sensitive to both Aβ(1–40) and Aβ(1–42), and this is reversed by co-treatment with Aβ(1–38). In contrast and somewhat counterintuitively, the *APOE* ε2/ε3 cell line does not respond to either Aβ(1–40) or Aβ(1–42), but a combination of the otherwise non-toxic Aβ(1–38) peptide with a subequimolar concentration of Aβ(1–42), but not Aβ(1–40), triggers a loss of mitochondrial respiration in this cell line. This was unexpected, but certainly reminiscent of our patch-clamping results wherein a subequimolar concentration of Aβ(1–42) leads to a loss of current density in hippocampal neuronal cultures treated with Aβ(1–38), which does not exert any significant effect on its own.

This notion of pools of truncated Aβ peptides in different relative proportions having different toxicity profiles is not new. For example, smaller aggregated species of soluble Aβ(1–42) are thought to exert deleterious effects by increasing changes in membrane permeability^47^, potentially through a direct interaction with phospholipids^35^, while larger aggregates are thought to trigger the pro-inflammatory reactions often associated with the AD brain^47^. Furthermore, soluble, primarily N-terminally truncated Aβ extracts from the AD brain can induce amyloidosis when injected intracerebroventricularly in mice, whereas soluble Aβ extracts from CSF (containing both C-terminally and N-terminally truncated species) do not^48^. Our Western blotting observations suggest that unlike the stable Aβ(1–42) aggregate we observe, any potential Aβ(1–38) aggregate is likely not stable under SDS-denaturing conditions. This inherent difference between aggregates of Aβ(1–38) and Aβ(1–42) is supported by the anomalous loss of ThT fluorescence we observed with Aβ(1–38) alone. Part of this could be explained by the fact that ThT, while a valid probe for monitoring protein folding and amyloid fibril behaviour, actually binds non-covalently (reversibly) to cross-β-strand structures, rather than to the β-sheet region of amyloid structure^49^. Thus, the ThT fluorescence associated with Aβ(1–38) in isolation may be depicting a dynamic flux in conformation of this peptide *over time*, whereas those traces associated with complex mixtures containing Aβ(1–38) may be depicting more stable conformations owing to Aβ variant interactions. Whatever the mechanism, it is clear that studying these peptides in isolation is biasing our understanding of their influence in pathophysiological mixtures.

The different stages of AD progression have been associated with different proportions of Aβ peptide in the RIPA/SDS-soluble *versus* the insoluble (plaque-associated) fractions^50^. We recently reported significant Aβ(1–40) and Aβ(1–42) levels in the guanidine-extractable, plaque-associated fraction in autopsy AD brain samples^27^ and we now reveal sex-dependent differences (with some influence of *APOE* ε*4* status) in levels of soluble Aβ peptides in these same samples. The levels of soluble Aβ(1–38), Aβ(1–40), and Aβ(1–42) were all increased in samples from donors with a diagnosis of EOAD, confirming a previous study based on aggressive, genetic forms of AD^12^ and cell-based studies of familial mutations in the gene encoding presenilin-1, the catalytic core of the γ-secretase complex^51^. This suggests an indiscriminate processing of the APP precursor through to a heterogeneous pool of Aβ peptides in these more aggressive cases of the disease. In contrast, there were increases in the levels of all three peptides in the LOAD cortical samples, but only Aβ(1–42) was significantly elevated over control levels. Although similar patterns emerged in the hippocampal samples, they were not statistically significant and, in fact, levels in female LOAD samples remained remarkably unchanged from those in control samples. These observations continue to support differences in the male and female LOAD brain, and indirectly support the temporal pattern of amyloid burden that has been associated with AD progression, *i.e.*, increases in amyloid in the cortex precede those in the hippocampus^52^. A temporal relevance to the interaction between Aβ(1–38) and Aβ(1–42) was confirmed by our *C. elegans* experiments, in which Aβ(1–38) was able to mitigate an Aβ(1–42)-mediated phenotype at earlier time-points, but any ‘protection’ was gradually lost as both peptides continued to accumulate.

The higher cortical Aβ(1–42)/Aβ(1–40) ratios in both male and female LOAD samples, and a higher cortical Aβ(1–42)/Aβ(1–38) ratio in males with LOAD, are in keeping with decreases in the CSF/plasma ratios of Aβ(1–42)/Aβ(1–40) or Aβ(1–42)/Aβ(1–38) aligning with disease progression or cognitive decline^5,9,16,53,54^ as well as with the increased Aβ(1–42) and Aβ(1–42)/Aβ(1–40) estimates from the corresponding insoluble, plaque-associated fractions of these samples^27^. Furthermore, the higher Aβ(1–42)/Aβ(1–40) and Aβ(1–42)/Aβ(1–38) ratios were correlated with earlier age-at-death in males, while a higher Aβ(1–42)/Aβ(1–40) ratio correlated with later age-at-death in females. We did not observe similar patterns in the corresponding hippocampal samples. These data remain cross-sectional and while they cannot inform on whether any observed changes were adaptive or causative, they certainly do support differences in the male and female AD brain, potentially suggesting differences in Aβ clearance mechanisms between the sexes, and supporting the consideration for different therapeutic strategies based on sex. We previously observed higher levels of Aβ(1–38) in older male (*vs.* female) J20 (APP_Swe/Ind_) mouse brains^13^ and potential sex-dependent differences in clearance (female > male) from the brain have been shown in the APP_Swe_/PS1ΔEx9 mouse^55^. Sex-dependent differences in CSF levels of Aβ(1–42) have been shown to correlate with differences in cognitive function, for example, based on the Mini-Mental State Examination (MMSE)^56^ or the Word List Delayed Recall^57^, while higher levels of plasma Aβ(1–42) have been detected in women with preclinical sporadic AD^58^.

γ-Secretase inhibitors capable of shifting the cleavage of APP to yield Aβ(1–38) at the expense of Aβ(1–42)^14^ could have translational relevance, yet it is important to note that shorter Aβ length variants are not necessarily all beneficial; for example, an increase in CSF levels of Aβ(1–34), a BACE1-mediated fragment^59^ found in AD CSF and brain extracts^9,48^, is a putative marker for conversion from mild cognitive impairment to AD^59^, while Aβ(1–24), the APP fragment ostensibly tied to MPP9 cleavage, can act as a seed for Aβ(1–42) fibrillogenesis and trigger behavioral and cognitive phenotypes in the wildtype mouse similar to those observed in an age-matched APP/PS1 mouse^60^.

In reality, the interaction of Aβ peptides is likely far more complicated than suggested herein. Indeed, our SPR results reveal that a subequimolar concentration of Aβ(1–38) *interferes* far more with the ability of Aβ(1–40) [*vs* Aβ(1–42)] to recognize Aβ(1–38) or Aβ(1–40), but that the same subequimolar concentration of Aβ(1–38) *promotes* the interaction between Aβ(1–40) and Aβ(1–42). These SPR results (which are reminiscent of our observation that Aβ(1–38) exerts opposite effects on Aβ(1–42)- and Aβ(1–40)-mediated ThT fluorescence) clearly expose complex interactions that are relevant to the emerging interest in understanding the clinical impact of a heterogeneous pool of Aβ variants.

It is often simplistically presumed that any Aβ length variant, or its accumulation, exacerbates the pathological progression associated with AD. Unfortunately, this misconception has underscored AD research for so long that evidence to the contrary, *e.g.* Aβ burden in cognitively intact elderly individuals or any beneficial roles reported for Aβ peptides, is often viewed as an anomaly^1^. Yet, the possibility that soluble Aβ length variants could be exerting a multitude of roles, with some being neuroprotective rather than ‘amyloidogenic’ and neurotoxic, would support a neurobiological ‘benefit’ for heterogeneity within the pool of Aβ peptides and could help to explain why the indiscriminate targeting of Aβ peptide(s) in AD clinical trials has met with a succession of negative outcomes^1,61^.

## Materials and methods

### Peptides and antibodies

Synthetic Aβ(1–38) (cat#: H-2966), Aβ(1–40) (H-1194), and Aβ(1–42) (H-1368) were obtained from Bachem Americas, Inc., and the amino acid composition was confirmed by mass spectrometry. All peptides were reconstituted in hexafluoroisopropanol (HFIP) so as to disrupt any preexisting β-sheet structures^24^ and residual HFIP was evaporated prior to peptide use in any assay. Different commercial lots were used to avoid the possibility that our results were biased by a particular batch of synthetic peptide(s).

The anti-β-amyloid antibody [clone 6E10: targets residues 1–16 of the Aβ peptide: cat# 803016] was obtained from BioLegend. The antibody raised against the C-terminal region of human APP695 [targets residues 676–695: cat# A8717] was obtained from Sigma-Aldrich Ltd. Protein-A/G sepharose was obtained from GE Healthcare Bio-Sciences Inc.

### Biophysical experiments

Atomic Force Microscopy (AFM) measurements were used to monitor changes in fibril morphology in our peptide preparations and were carried out on a PicoSPM instrument (Molecular Imaging) operating in intermittent contact mode. A silicon cantilever (NSG_L, K-TEK Nanotechnology) with tip curvature of radius < 10 nm, a force constant of approximately 58 N/m, and a resonant frequency of approximately 190 kHz was used for each measurement. Experiments were conducted at a set-point ratio of approximately 0.8–0.85 from the free-amplitude of the cantilever and all measurements were obtained in a vibration isolation system. The scan rate was 0.5–1.0 Hz (512 pixels *per* line) for all images. Data were analyzed using SPIP V5.1.6 software (Image Metrology).

Mica surfaces were prepared by applying 25 µL of poly-L-lysine (0.01% 70–150 kDa) for 3 min. Surfaces were then rinsed three times with Millipore water and gently dried under nitrogen gas. Aβ peptide solutions (0.1 mg/mL in PBS) were incubated for 0, 24, or 48 h and then deposited onto the freshly coated surfaces for 3–5 min, rinsed with water, air dried, and stored in a dust-free environment until imaged.

Gel electrophoresis (Western blotting) was used to visualize the aggregation potential of Aβ peptide mixtures. Peptide solutions were incubated at room temperature for 24 h. Aliquots were resolved using either standard 15% SDS-PAGE or a discontinuous 8 M urea/12% SDS-PAGE system as we have done for Aβ peptides isolated from the insoluble (guanidine-extractable) fraction of these same tissues^27^ and then transferred to nitrocellulose membrane. We found that boiling the membrane was critical for detection of the monomeric Aβ peptides (urea gel electrophoresis), but hindered the detection of the higher molecular weight Aβ aggregates (and thus was avoided for those blots). Membranes were blocked in TBS containing 1% BSA and probed overnight (4 °C) with the 6E10 antibody.

Thioflavin T (ThT) fluorescence is thought to reflect binding of ThT dye to putative β-sheet structures associated with amyloid fibril formation^62^. In situ ThT (Acros Organics) fluorescence measurements were performed on a Bio-Rad CFX96 Thermocycler operating at 25 °C. Triplicate solutions (20 µL) of the Aβ peptides (20 µM in PBS, pH 7.4) were incubated with 10 µM ThT. Measurements were obtained every 60 s using λex = 450–490 nm and λem = 510–530 nm. A control solution containing only 10 µM ThT was subtracted from all test measurements.

The analysis of protein secondary structure using Circular Dichroism (CD) spectroscopy is based on the differential absorption of polarized light by optically active molecules. CD measurements were carried out on a Pistar-180 CD spectrometer (Applied Photophysics Ltd.) at 25 °C using a 0.1 cm optical path-length quartz cuvette. Aβ solutions were scanned from 260–190 nm in 0.5 nm steps at a scan rate of 5 nm/min and a bandwidth of 6 nm. The CD spectrometer was calibrated with 10-camphorsulphonic acid and spectra were background-subtracted using PBS, pH 7.4. Depicted CD spectra are the average of three spectra smoothed using a five-point Savitsky-Golay smoothing algorithm^63^. Deconvolution was performed using BeStSel^64^.

Dynamic Light Scattering (DLS) determines the size distribution of particles based on the proportion of incident light scattered, *e.g.* the larger the particles, the greater the scattering. DLS measurements were carried out in a quartz cuvette (Hellma Analytics) on a Dyna-Pro MS800 instrument (Wyatt Technologies) at 25 °C using an 824.8 nm (55 mW) laser diode. Scattered light was collected at 90° with an Avalanche photodiode detector. Data were acquired for 5 s and analyzed with DYNAMICS software (Wyatt Technologies).

Surface Plasmon Resonance (SPR) is a cell-free, optical technique used for monitoring real-time molecular interactions between a ligand immobilized on the surface of a flow cell and an injected analyte solution. SPR experiments were performed on a Proteon XPR36 (Bio-Rad) instrument. Standard amine-coupling chemistry was used to immobilize Aβ peptides onto a GLC sensor chip^65^. Briefly, sensor surfaces were activated using a solution of 20 mM 1-Ethyl-3-(3-Dimethylaminopropyl) carbodiimide: 5 mM sulfo-N-hydroxysulfosuccinimide injected for 5 min at 30 µL/min. After activation, individual Aβ peptides (50 µg/mL in 10 mM acetate buffer, pH 4.0–4.5) were injected at 25 µL/min for 5 min, after which any unoccupied succinimide sites were deactivated with an injection of ethanolamine (1 M, pH 8; 5 min, 30 µL/min). A reference surface was generated in the absence of Aβ peptide(s). The analyte solution was injected over the flow cell at 5 µL/min (120 s) and then replaced with wash buffer (20 min); any interaction between the test and immobilized proteins yields a sensorgram with association and dissociation phases.

### Functional experiments

Mitochondrial metabolic activity based on the MTT conversion assay was used as an index of cell viability. The immortalized mouse hippocampal HT-22 cell line^66^ was cultured in DMEM/low glucose medium containing 10% fetal bovine serum (FBS). Human skin fibroblasts from female in-patients without metabolic disease were obtained from the Montreal Children’s Hospital Cell Repository and have been characterized elsewhere^67^. The fibroblasts were maintained in DMEM/high glucose medium containing 10% FBS.

Cells (10,000/well) were treated with Aβ peptides (24 h) and the conversion of 3-(4,5-dimethylthiazol-2-yl)-2,5-diphenyltetrazolium bromide (MTT; 0.5 mg/mL; 2 h; 37 °C; 5% CO_2_) to the formazan product was quantified by spectrophotometry (absorbance = 570 nm)^68^.

Electrophysiological paradigms, *e.g.* LTP (synaptic plasticity) and whole cell patch-clamping (membrane conductance) were used to assess the functional impact of Aβ peptide mixtures.

*LTP in acute hippocampal slice preparations*: These procedures were approved by Memorial University’s Animal Care Committee (PI: MPP). Four- to six-week old male C57BL/6 mice (Charles River Laboratories, Inc) were anesthetized using isoflurane and brains were quickly removed into ice-cold oxygenated slicing solution (in mM): 125 NaCl; 2.5 KCl; 25 NaHCO_3_; 1.25 NaH_2_PO_4_; 2.5 MgCl_2_; 0.5 CaCl_2_; and 10 glucose^69^. Transverse hippocampal slices (350 µm) were immediately transferred to artificial cerebrospinal fluid (aCSF), which had the same formulation as the slicing solution except for MgCl_2_ (1 mM) and CaCl_2_ (2 mM). Slices were allowed to recover (90 min, RT) and then transferred to a recording chamber. Oxygenated aCSF was continuously perfused at a flow rate of 1–2 ml/min (25 °C). Glass pipettes were pulled using a Narishige PB-7 pipette puller to a resistance of 1–3 MΩ when filled with aCSF.

Stimulation was applied to the Schaffer collaterals through a glass pipette using an Iso-flex stimulator and field excitatory postsynaptic potentials (fEPSPs) were recorded by placing a glass recording electrode in the CA1 *stratum radiatum*, approximately 400 µm from the stimulating electrode. A quick input/output plot was generated for each individual slice by increasing the stimulation intensity and an intensity that elicited 30–40% of maximum slope was used for the experiment. A stable baseline was established using 0.1 ms pulses at a frequency of 0.33 Hz before bath-applying Aβ(1–42) or Aβ(1–38), either alone or in combination, for 20 min before LTP induction, which consisted of a standard theta burst stimulation protocol of 10 bursts of 4 pulses at 100 Hz, with 200 ms interburst intervals. Aβ application continued for 5 min after LTP induction. Recordings continued for 60 min after LTP induction and the percent potentiation was analyzed as the average percent increase in the initial 1–2 ms of the fEPSP slope for the last 5 min of the experiment (55–60 min post-induction) compared to the 10 min of stable baseline prior to induction. All data were collected and analyzed using pClamp 10 software (Molecular Devices) and GraphPad PRISM.

*Whole Cell Patch-Clamp Electrophysiology in isolated hippocampal neurons*: These protocols conformed to the guidelines approved by the President's Committee on Animal Care, University of Regina (PI: JB). One-year old mice were sacrificed by pentobarbital overdose (120 mg/kg) and hippocampi were removed to PBS containing 4% Penicillin–Streptomycin and dissociated in sterile collagenase solution (1 h; 37 °C)^70^. Isolated neurons were plated onto Matrigel-coated coverslips and astrocyte-conditioned medium was provided to increase concentration of growth factors. Cultures were maintained at 37 °C in humidified 5% CO_2_.

Nystatin-perforated patch recordings were made using an AxoPatch 200B, and signals were filtered with a low-pass 5 kHz filter, digitized (Digidata 1550 series) and analyzed using Clamfit 10.7 software (Molecular Devices) as previously described^71^. The series resistance was compensated and junction potentials were cancelled in all experiments. Cells were held at − 60 mV, step depolarized to the indicated test potential (between − 80 and + 100 mV in 20 mV increments) for 50 ms, and sampled at a frequency of 1000 Hz. Patch pipettes were pulled (PC-10, Narishige International) to a resistance of 6–8 MΩ when filled with an internal pipette recording solution containing (in mM): 135 KCl; 5 NaCl; 2 CaCl_2_; 10 HEPES (pH 7.2), and nystatin (250–500 µg/mL). The extracellular recording solution contained (in mM): 135 NaCl; 5 KCl; 2 CaCl_2_; 2 MgCl_2_; 10 D-glucose; 10 HEPES (pH 7.4). The hippocampal neurons were treated with Aβ(1–42) or Aβ(1–38) alone or in combination, and current density (*e.g.* current normalized to the cell capacitance) 24-h post-exposure to Aβ peptides was measured at 0 and 20 mV, 100 ms after the voltage step, in terms of mean I–V curves of steady-state currents.

The in vivo influence of the Aβ(1–38) peptide on Aβ(1–42) phenotype was assessed using the *Caenorhabditis elegans* (*C. elegans*) worm.

A *Nhe*I/*Sac*I Aβ(1–38) PCR insert was generated using the Aβ(1–42) transgene (*dvIs100*) in the GMC101 strain^28^ as a template and substituting GTT (Val39) with TGA (stop codon), thereby eliminating codons 39–42. The *Nhe*I/*Sac*I Aβ(1–38) fragment was then cloned downstream of the *Punc-54* enhancer in pPD30.38 to generate pCEC-DM-AB38 (*e.g.* carrying the *Punc-54::A*β*(1–38)* transgene).

The *C. elegans* strains were maintained on Normal Growth Medium agar plates at 20 °C^72^, unless otherwise specified. The following strains were used: GMC101 [*dvIs100* (*Punc-54::A*β*(1–42)::unc-54 3′UTR; Pmtl-2::GFP*)], CL2122 [*dvIs15* (*pPD30.38; Pmtl-2::GFP*)], CEC220 [*sasEx45* (*Punc-54::A*β*(1–38)::unc-54 3′UTR; Podr-1::DsRed*)], CEC222 (*dvIs100; sasEx45*). CEC220 was generated by microinjection of a 20 ng/µL mixture of pCEC-DM-AB38 and *Podr-1::DsRed* as a co-injection marker. To generate CEC222, GMC101 males were crossed to CEC220 hermaphrodites, and homozygous *dvIs100* F2 worms segregating the *sasEx45* chromosomal array were isolated and maintained. To test CEC220 [Aβ(1–38)] and CEC222 [Aβ(1–42); Aβ(1–38)] strains, synchronized worms showing DsRed in head neurons were first identified and isolated under a Nikon SMZ1500 stereomicroscope.

The GMC101 strain^28^ expresses the AD-related Aβ(1–42) under the control of the muscle-specific *unc*-54 (heavy chain muscle myosin) enhancer, and switching these worms from 20 to 25 °C triggers intracellular accumulation of Aβ(1–42) within the body wall muscle, eventually leading to paralysis^73^. Such defects in movement can be quantified using ‘thrashing behaviour’, *e.g.* the number of complete bends of the dorsal or ventral side of the animal. Basically, a worm is transferred to a drop of M9 buffer, allowed to acclimate (20 s), and then the number of body bends in a 30-s test period is recorded manually. Thus, the thrashing rate can be used as a proxy for compromised muscle function due to Aβ accumulation. Each test group relied on a minimum of 60 worms *per* time point.

The translational potential of our observations was inferred by determining soluble Aβ peptide levels in autopsied human brain (frontocortical and corresponding hippocampal samples) from 26 controls (12 M/14 F), 16 early-onset/EOAD (*i.e.*, age of onset < 65 years: 7 M/9 F), and 18 late-onset/LOAD (*i.e*., age of onset 65 + years: 8 M/10 F) cases. The samples were obtained from the Douglas-Bell Canada Brain Bank (McGill University) and diagnoses were confirmed histopathologically by on-site pathologists. These studies were conducted according to the University of Saskatchewan Policies and Procedures for Ethics in Human Research and are covered by the Research Ethics Office Certificate of Approval ‘Bio 06–124′ (PI: DDM).

We used our published protocol to isolate *soluble* Aβ peptides^13,74^. Briefly, samples (20–30 mg wet weight) were homogenized in 20 volumes of ice-cold RIPA buffer containing a protease inhibitor cocktail and centrifuged at 12,000×*g* (10 min; 4 °C). The supernatants (300 µg input protein) were immunodepleted of full-length (FL)-APP using a C-terminally-directed antibody and then immunoprecipitated for Aβ peptides using the 6E10 antibody; the 6E10-immunocomplexes were resolved by urea gel electrophoresis and transferred to nitrocellulose membranes, which were boiled for 5 min, blocked in milk casein (1 h, RT), and probed for the Aβ peptides (6E10 antibody). Detection relied on a goat-anti-mouse AlexaFluor-conjugates scanned in the 680 nm channel and a LI-COR imaging system. Densitometry was performed using the Image Studio Lite Western Blot Quantification software preloaded on the imaging system. Any processing (*e.g*., change in contrast setting) was applied equally across each and every image in its entirety. Every blot/image included controls (neurologically normal cases).

## Statistics

The data were analyzed using non-parametric statistics, *i.e.* the Mann–Whitney U test; ANOVA (Kruskal–Wallis) with adjustment for multiple comparisons using Dunn's post hoc test; or two-way ANOVA with a Dunn–Šidák correction for post hoc analysis (GraphPad Prism). Significance was set at *P* < 0.05. Analyses in which *P* values fell between 0.05 and 0.1 were discussed as *tendencies*. Possible bias was minimized by having our co-authors perform the respective protocols with only minimal knowledge of the test hypothesis and without any a priori knowledge of the outcomes of other collaborations described herein. Any possibility of bias using our autopsy-derived data was mitigated by having some individuals assay de-identified samples and having other individuals perform the analysis, *i.e.* scanning/densitometry.

## Supplementary Information

Supplementary Information 1.
