# Supplementary material for: The Aβ(1–38) peptide is a negative regulator of the Aβ(1–42) peptide implicated in Alzheimer disease progression
Source: Sci Rep. 2021 Jan 11;11:431. doi: 10.1038/s41598-020-80164-w (PMC7801637; doi:10.1038/s41598-020-80164-w)
Supplement: Supplementary file 1 — Supplementary Information 1. [file 41598_2020_80164_MOESM1_ESM.pdf]

The Aβ(1-38) peptide is a negative regulator of the Aβ(1-42) peptide implicated in Alzheimer disease progression

Maa O. Quartey[1], Jennifer N. K. Nyarko[1], Jason M. Maley[2], Jocelyn R. Barnes[3], Maria A. C. Bolanos[4], Ryan M. Heistad[1], Kaeli J. Knudsen[1], Paul R. Pennington[1], Josef Buttigieg[4], Carlos E. De Carvalho[5], Scot C. Leary[6], Matthew P. Parsons[3], Darrell D. Mousseau[1,*]

[1]: Cell Signalling Laboratory, Department of Psychiatry, University of Saskatchewan, Saskatoon, SK, Canada;

[2]: Saskatchewan Structural Sciences Centre, University of Saskatchewan, Saskatoon, SK, Canada;

[3]: Division of BioMedical Sciences (Neurosciences), Memorial University of Newfoundland, St. John's, NL, Canada;

[4]: Department of Biology, University of Regina, Regina, SK, Canada;

[5]: Department of Biology, University of Saskatchewan, Saskatoon, SK, Canada;

[6]: Department of Biochemistry, Microbiology and Immunology, University of Saskatchewan, Saskatoon, SK, Canada.

*Address correspondence to: D.D. Mousseau, Cell Signalling Laboratory, GB41 HSB, University of Saskatchewan, 107 Wiggins Rd., Saskatoon, SK (S7N 5E5), Canada.

e-mail: darrell.mousseau@usask.ca

Running title: Aβ38 is a negative regulator of Aβ42

List of supplementary figures (uncropped Western blots)

Supplementary Figure 1. A scan of three blots used for our Aβ peptide co-incubation experiment (cropped and used for Figure 2).

Supplementary Figure 2. A scan of a blot used to identify Aβ peptides in *C. elegans* extracts (one light; one overexposed to see the putative Aβ(1-38) dimer).

Supplementary Figure 3. A representative full-length blot depicting soluble Aβ peptides isolated from the RIPA fraction of human cortical brain samples (cropped for Figure 7).

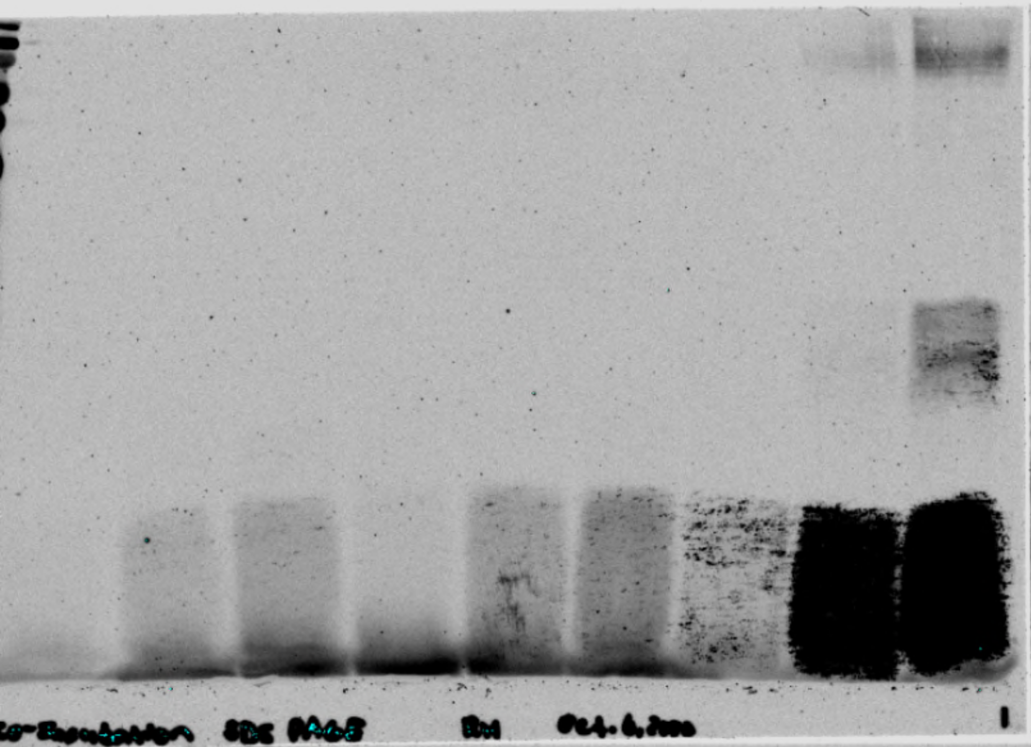
SDS PAGE
Oct. 6, 2020

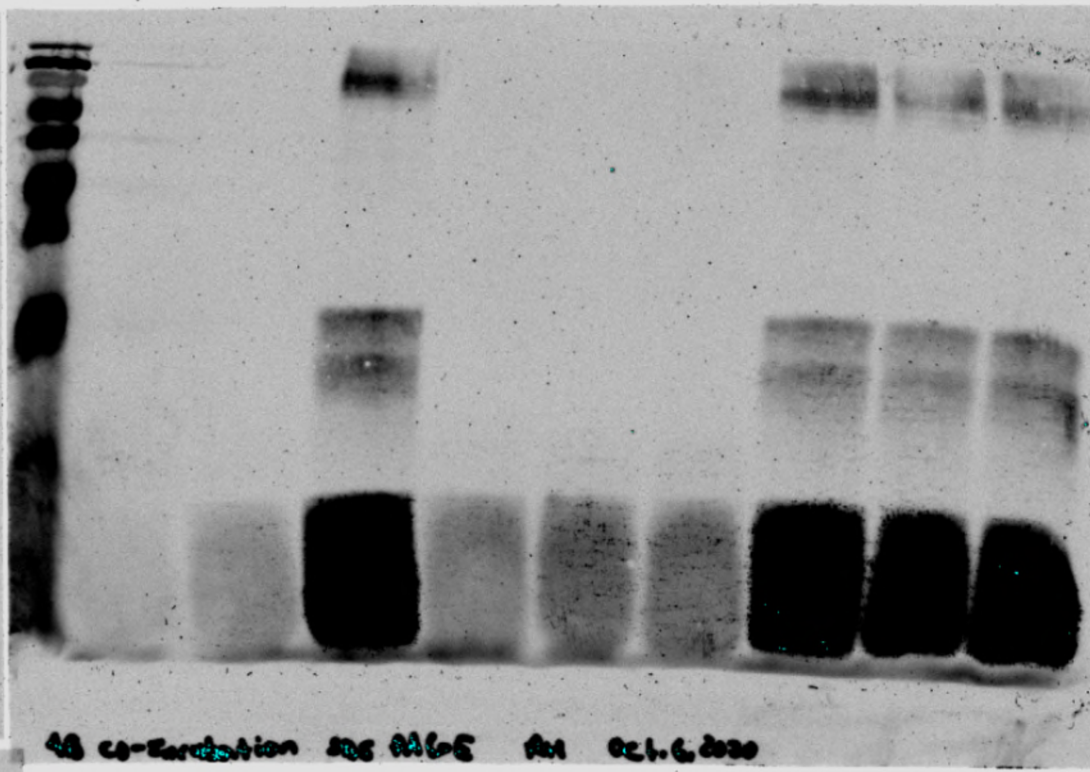
SDS PAGE
Oct. 6, 2020

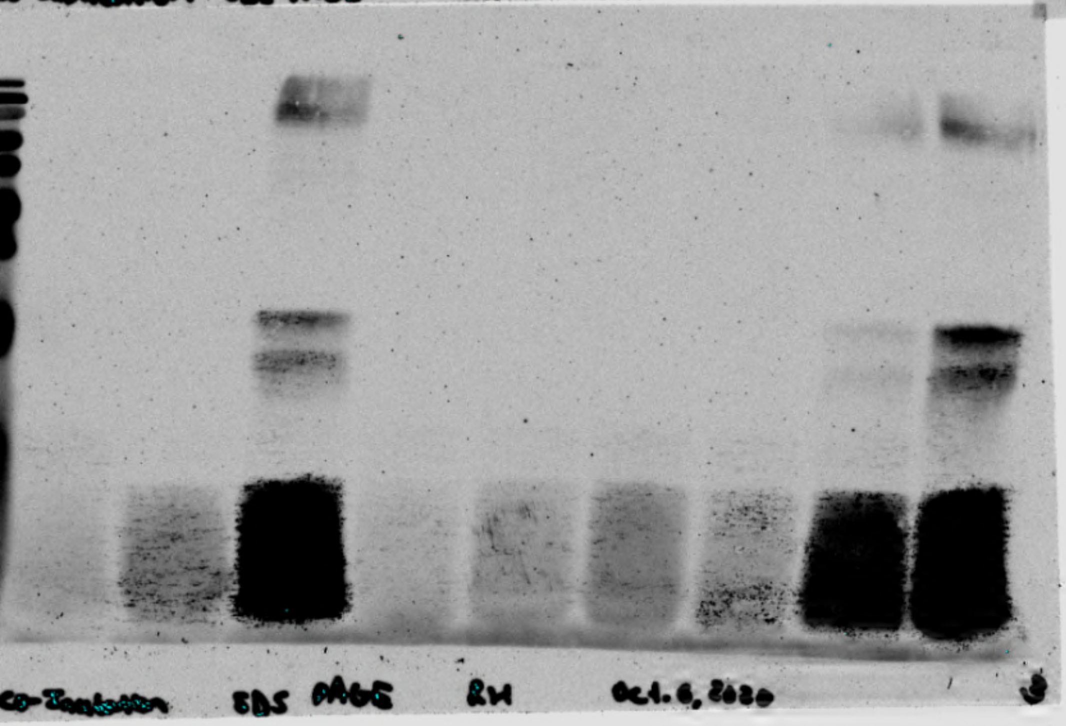
SDS PAGE
Oct. 6, 2020

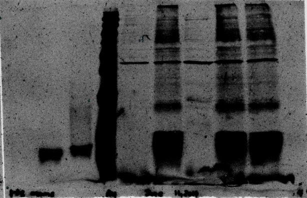

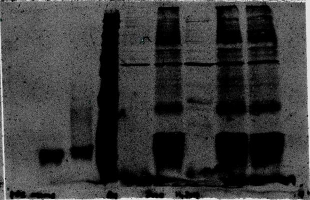

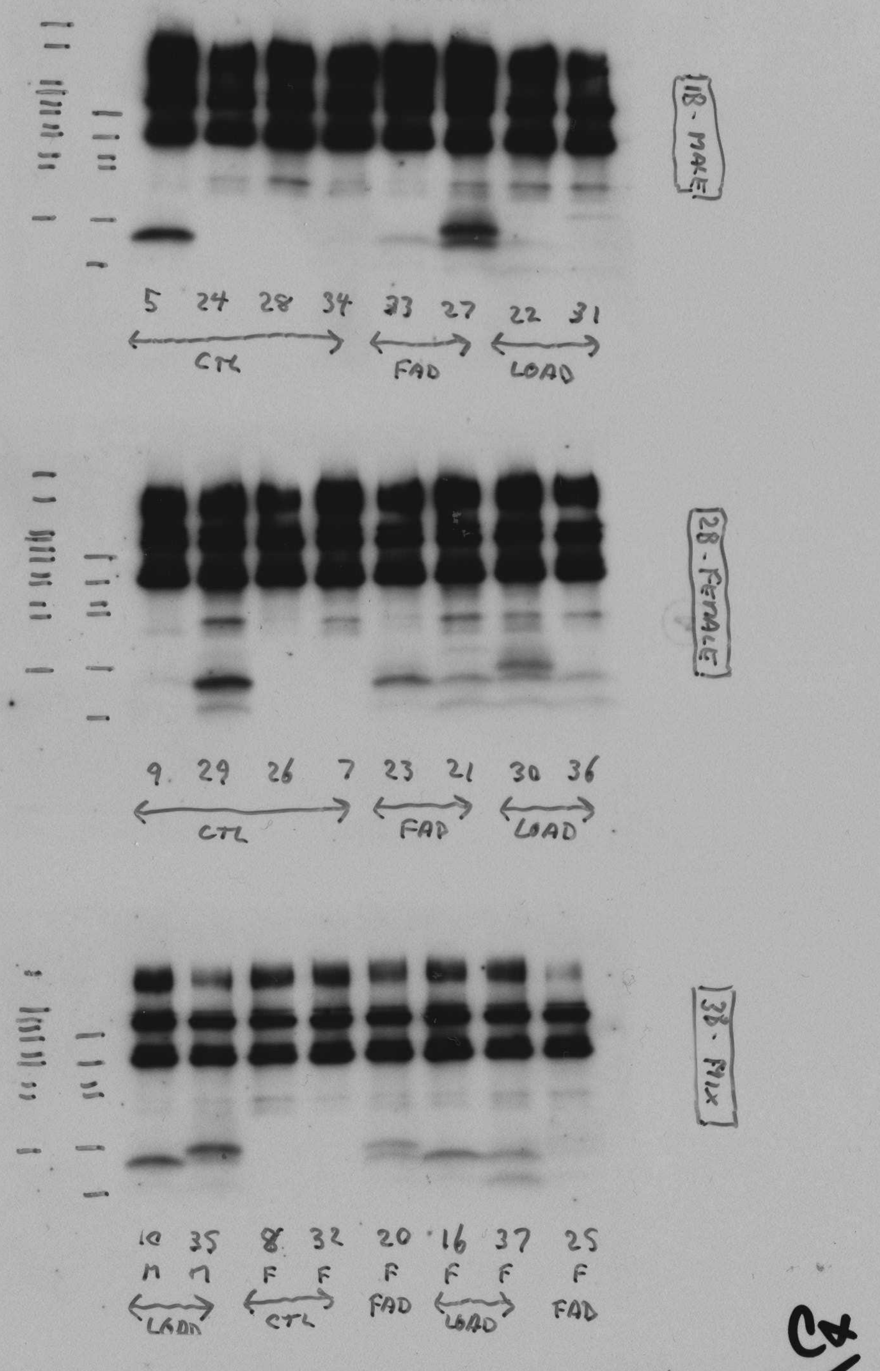
1B - MALE
5 24 28 34 23 27 22 31
CTL
FAD
LOAD
2B - FEMALE
9 29 26 7 23 21 30 36
CTL
FAD
LOAD
3B - MIX
10 35 8 32 20 16 37 25
M M F F F F F F
LOAD
CTL
FAD
LOAD
FAD
